# Breast Cancer Mammograms Classification Using Deep Neural Network and Entropy-Controlled Whale Optimization Algorithm

**DOI:** 10.3390/diagnostics12020557

**Published:** 2022-02-21

**Authors:** Saliha Zahoor, Umar Shoaib, Ikram Ullah Lali

**Affiliations:** 1 Computer Science Department, University of Gujrat, Gujrat 50700, Pakistan; umar.shoaib@uog.edu.pk; 2 Information Sciences Department, University of Education Lahore, Jauhrabad Campus, Khushab 41200, Pakistan; m.i.lali@ue.edu.pk

**Keywords:** breast cancer, classification, deep learning, features fusion, features optimization

## Abstract

Breast cancer has affected many women worldwide. To perform detection and classification of breast cancer many computer-aided diagnosis (CAD) systems have been established because the inspection of the mammogram images by the radiologist is a difficult and time taken task. To early diagnose the disease and provide better treatment lot of CAD systems were established. There is still a need to improve existing CAD systems by incorporating new methods and technologies in order to provide more precise results. This paper aims to investigate ways to prevent the disease as well as to provide new methods of classification in order to reduce the risk of breast cancer in women’s lives. The best feature optimization is performed to classify the results accurately. The CAD system’s accuracy improved by reducing the false-positive rates.The Modified Entropy Whale Optimization Algorithm (MEWOA) is proposed based on fusion for deep feature extraction and perform the classification. In the proposed method, the fine-tuned MobilenetV2 and Nasnet Mobile are applied for simulation. The features are extracted, and optimization is performed. The optimized features are fused and optimized by using MEWOA. Finally, by using the optimized deep features, the machine learning classifiers are applied to classify the breast cancer images. To extract the features and perform the classification, three publicly available datasets are used: INbreast, MIAS, and CBIS-DDSM. The maximum accuracy achieved in INbreast dataset is 99.7%, MIAS dataset has 99.8% and CBIS-DDSM has 93.8%. Finally, a comparison with other existing methods is performed, demonstrating that the proposed algorithm outperforms the other approaches.

## 1. Introduction

Cancer is a fatal disease, with an estimated ten million deaths and 19.3 million cancer cases reported in 2020 [1]. Breast cancer after lung cancer is the second utmost common cancer [2], and a fifth foremost reason of death in women [2,3]. In 2020, 684,996 deaths occurred with breast cancer and 2.3 million new cases diagnosed in women (https://gco.iarc.fr/today/data/factsheets/cancers/20-Breast-fact-sheet.pdf) (accessed on 20 October 2021) [1]. In less developed countries the breast cancer is the foremost cause of death [4,5]. The cells in the breast tissues change and split into multiple cells, causing a mass or lump. Cancer begins in ducts or lobules that are connected to the nipples (https://www.cancer.org/content/dam/cancer-org/research (accessed on 11 November 2021)) [3]. Most masses in the breast are benign that is, noncancerous, and cause fibroids, tenderness, area thickening, or lumps [3]. Mostly, breast tumors have no signs when small in size and can be easily treated (https://www.cancer.org/content/dam/cancer-org/research (accessed on 11 November 2021)). Painless mass is the sign of abnormal cells. Family history, reproductive factors, personal characteristics, excess body weight, diet, alcohol, tobacco, environmental factors, and other risk factors, such as night shift work, are all breast cancer issues. In primary phase, breast cancer spreads slowly but with passage of time it affect to other body parts.(https://www.cancer.org/content/dam/cancer-org/research (accessed on 11 November 2021)).

Many tests are recommended for the diagnosis of breast tumors, including mammography, magnetic resonance imaging (MRI) [6], ultrasound [7], and digital breast tomosynthesis (https://www.cancer.org/content/dam/cancer-org/research (accessed on 11 November 2021)) [1]. The most recommended test at an early stage is mammography. The mammography is an affordable, low radiation test that is suggested for early diagnosis of the breast tumor [1,8]. The MRI is an alternative test that is used to confirm the presence of a tumor. An allergic reaction to the contrast dye may occur during the MRI test. This is an unintended consequence of the MRI test. At an initial phase, the recommended test is mammography.In an initial phase, treatment of breast cancer is possible [8]. There are many treatment methods such as surgery to remove the defected area, medication, radiation therapy, chemotherapy, hormonal therapy, and immunotherapy (https://www.cancer.org/content/dam/cancer-org/research (accessed on 11 November 2021)) [2]. These treatments, when administered early on, have the potential to save lives. The survival rate is 90% in developed countries, 40% in South Africa and 66% in India if detected in an initial stage. The low-income countries have fewer resources, so early diagnosis methods and treatments can be helpful to save women’s lives (https://www.who.int/news-room/fact-sheets/detail/breast-cancer (accessed on 18 November 2021)) [8].

In medical imaging diagnosis of breast cancer has been extensively used. In the initial stage detection of breast tumors is critical. Mammography is the recommended procedure for early detection (https://www.cancer.org/content/dam/cancer-org/research (accessed on 11 November 2021)). In the diagnostic centers, more than one radiologist is present to diagnose the mammogram breast tumor. The radiologists accomplished single readings with or without CAD systems, as well as double readings in a known or non-known manner. For accurate results, a double reading is recommended for confirmation. In Dutch, double reading is the quality of practice. In double reading, the radiologists can classify the results into different types and stages. It can generate false-positive results [9]. To reduce burden of human observers and to minimize the false negative results many hospitals implemented double reading. Double reading is not an appropriate procedure due to cost and time constraints [10,11]. The results can be inaccurate due to two readings. The false-positive rates can be high, so there is a need to precisely diagnose abnormal regions and classify them into malignant or benign.In medical field, CAD systems always required to support the radiologists as a second opinion.To reduce the perceptual errors CAD systems have been investigated. The computerized methods are used in CAD systems to detect image anomalies and perform tests. Human perception and decision-making abilities are aided by CAD systems. The medical diagnostician makes the final decision. CAD systems help radiologists detect and differentiate between normal and abnormal tissues [8,11,12]. The masses are symptoms of breast tumors. The masses are benign and malignant. The benign are round or oval-shaped, while the masses are round or irregular in shape. The most whitened area in mammogram images is mass [5,8]. Mammogram images have a complex structure, making it difficult for radiologists to extract features and precisely classify the images. Many researchers have introduced numerous methods for feature extraction and classifying diseases, but these methods still need to be improved.

Different deep learning models are available that perform different tasks such as object detection [13], visual tracking [14], semantic segmentation [15], and classification [16]. The researchers proposed different models like AlexNet, GoogleNet, ResNet, MobileNet, and EfficientNet to perform the classifications [17]. 

In this research study, Features are extracted to achieve the best performance of the model by using Fine-tuned MobilenetV2 and Fine-tuned Nasnet Mobile. The Modified Entropy Whale Optimization Algorithm (MEWOA) is applied to improve the features. The performance speed of the system will be increase by minimizing the computation cost. The selection of features is performed. The feature selection methods will increase the performance of the model by decreasing the computational cost and the load of the classification. The feature selection technique is used to choose the model’s most relevant features that will improve the model’s accuracy. The feature fusion is performed to get the internal information of the multiple input data by joining it into a single feature vector. Feature fusion helps to join the multiple data into a single place.

In this paper, three datasets are taken. Data augmentation is performed to increase the images. The Modified Entropy controlled Whale Optimization Algorithm (MEWOA) is proposed for optimal features selection. The major contributions are as follows:

- Data augmentation is performed using three mathematical formulas: horizontal shift, vertical shift, and rotation 90.
- Two deep learning pre-trained models are fine-tuned such as Nasnet mobile andMobilenetV2 and deep features are extracted from the middle layer (average pool) instead of the FC layer.
- We proposed a Modified Entropy-controlled Whale Optimization Algorithm for optimal feature selection and reducing the computational cost.
- We fused the optimal deep learning features using a serial-based threshold approach.

### Literature Review

To perform the feature extraction and classification lot of models have been proposed [18]. To extract the features and to perform mammogram images classification into malignant and benign CAD is developed by deploying the Deep Convolutional Neural Network (DCNN) and AlexNet model [19]. The SVM approach is used to connect to the last layer, which is fully connected to achieve good accuracy. Fine-tuning is also performed. The AUC is 0.94% and accuracy is 87.2% achieved [20]. The DCNN model is used for mammogram images detection. The model is fine-tuned [21]. To classify the malignant and non-malignant images, a CAD system is proposed. The K-Clustering technique and SVM classifier is used. Sensitivity and specificity of 96% are achieved by using the dataset DDSM [22]. The researcher took INbreast and CBIS-DDSM datasets in the form of png, resizing of the images is performed. The VGG and Resnet methods used for classification. The CBIS-DDSM achieved 0.90 AUC and 0.98 on INbreast dataset [23]. The Deep Learning models VGG, Resnet, Xception are applied to the CBIS-DDSM dataset. Transfer learning and fine tuning methods used to adjust the overfitting problem. The 0.84 AUC value is achieved on CBIS-DDSM [24]. To perofrm the classification researchers proposed Multi-View Feature Fusion(MVFF) method on mini-MIAS and CBIS-DDSM dataset. The AUC 0.932% is achieved [25]. The researchers used the MobilenetV2 model and performed transfer learning on the CBIS-DDSM dataset to perform the classification. The 74.5% accuracy is achieved. The data resizing and augmentation are performed [26]. The multi-level thresholding and radial region-growing methods are used on the DDSM dataset with an accuracy of 83.30% and an AUC of 0.92, which reduce the false positive rates [27].The CAD system is proposed by using the DDSM and mini-MIAS datasets. The histogram regions are used for segmentation and classification. K-means analysis is used to segment the images. The shape and texture features extracted and SVM classifier is used to perform the classification. The classification accuracy in mini-MIAS is 94.2% with an AUC 0.95% and in CBIS-DDSM 90.44% accuracy with an AUC value of 0.90% is achieved [28].

The Fuzzy Gaussian Mixture Model (FGMM) is used to classify the mammogram DDSM images. The FGMM achieves 93% accuracy, 90% sensitivity, and 96% specificity [29].

The CAD system is proposed to classify the INbreast dataset. To perform the classification the deep CNN model is used. The 95.64% accuracy, 94.78% AUC and 96.84% F1-score is achieved [30]. In the other study, the Modified VGG (MVGG) model is used to classify data from the CBIS-DDSM dataset. The hybrid transfer learning fusion approach is used in MVGG and ImageNet models. The modified MVGG achieves 89.8% accuracy, while MVGG and Imagenet combined by the fusion method achieve 94.3% accuracy [31]. In the other study, the researchers extract the features by using the Maximum Response (MR) filter bank that is convolved by the CNN to perform the classification. The fusion approach is applied to address the mass features. The accuracy on the CBIS-DDSM dataset after the fusion reduction approach is 94.3%, an AUC is 0.97%, and the specificity is 97.19% is achieved [32]. To extract features ensemble transfer learning approach is used.The neural networks are used to perform the classification. The 88% accuracy and an AUC 0.88% achieved on CBIS-DDSM [33]. To generate the ROI and classification of the INbreast dataset, a CAD system is proposed. Deep learning techniques such as a Gaussian mixture model and deep belief network are proposed. The cascade deep learning method is used to reduce the false-positive results. Bayesian optimization is performed to learn and segment the ROIs. In the last, the deep learning classifier is used to classify the INbreast images by achieving an accuracy of 91% and AUC is 0.76% [34].

The transfer learning [13] approach is used for improving the efficiency of the training models that are used to perform the classification. This approach makes learning faster and easier. Transfer learning is helpful when data is not available in a large amount. Transfer learning with fine-tuning is usually faster and training is easier when initialized the weights. It quickly learned transfer features using a small number of [33,34,35,36]. The transfer learning approach with CNNs has been used to classify the different types of images like histological cancer images, digital mammograms, and chest-X ray images [37].

To classify the INbreast and DDSM datasets, deep learning models CNN, ResNet-50, Inception-ResNetV2 were used. Mammogram images are classified as benign or malignant.The INbreast dataset achieved an accuracy of 88.74%, 92.55%, and 95.32% [38]. In another study, Faster-RCNN is used to detect and perform classification on the INbreast and CBIS-DDSM datasets. An AUC of 0.95 on the INbreast dataset is achieved [39]. The large number of data set require to train the deep learning models, so augmentation on the mini-MIAS dataset is performed by using the rotation and flipping method. The 450,000 images of MIAS after augmentation are taken and resized into 192 × 192. The images are classified into three categories using the multiscale convolutional neural network method (MCNN): normal, benign, and malignant. The AUC is 0.99 and the sensitivity is 96% [40]. The random forest (RF) on CNN with a pre-training approach is used to extract the hand-crafted features from the INbreast dataset. The 91.0% accuracy is achieved [41]. The author’s used physics informed neural network (PINN) by applying regression adaptive activation functions to predict the smooth and discontinuous functions to solve the linear and non-linear differential equation. To provide the smooth solution the nonlinear Klein Gordon equation has been solved, to use the high gradient solutions the non-linear Burgers equation and the Helmholtz equation, in particular, are used. To achieve the network best performance the activation function hyper parameter is optimized by changing the topology loss function that participates in the optimization process. To improve the convergence rate during initial training and solution accuracy the adaptive activation function outperforms in terms of learning capabilities. The efficiency can be increased by using this method [42]. To improve the performance of PINN the adaptive activation function use layer-wise and neuron-wise approaches. To complete the local adaption of activation function the scalable parameter is initialized in each layer of layer-wise and neuron to perform the optimization updation by utilizing the stochastic gradient descent algorithm. To increase the training speed the slope-based activation with loss function is applied [43]. The adaptive activation functions are utilizd to propose the Kronecker neural networks (KNNs). The number of parameters in the large network is reduced by KNNs by using the Kronecker product. The KNNs tempts faster loss decay as compare to feed forward network. For KNNs, the global convergence of gradient descent is established. The Rowdy activation function remove the saturation region with training parameters by using sinusoidal fluctuations [44].

## 2. Methods and Materials

**Figure 1:**
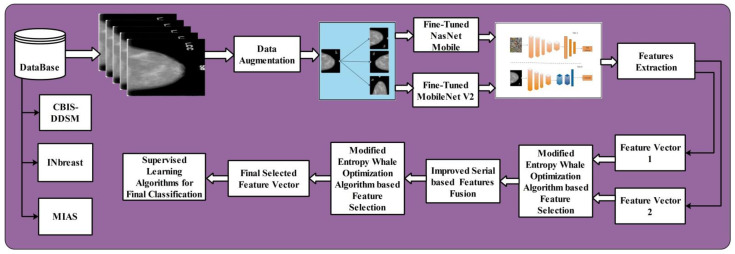
Proposed architecture of classification of breast cancer using deep learning.

This section illustrates the proposed methodology. The six steps are involved in the proposed methodology. In the first step, to increase the number of training samples the data augmentation is applied. In the second step, fine-tuning is performed on two selected deep models: MobilenetV2 and Nasnet mobile. Fine-tuned models are used to extract the features from the global average pool layer. In the third step, a Modified Entropy Whale Optimization Algorithm (MEWOA) is applied to the extracted deep features. In the fourth step, features are fused using a serial-based non-redundant approach. In fifth step, again, to reduce thecomputational time MEWOA is applied, and finally, classification is performed using machine learning classifiers. Figure 1 shows the detailed architecture of the proposed method. The detail of each step is given below.

### 2.1. Datasets

In this work, three publicly available mammography datasets are utilized for the experimental process: CBIS-DDSM [45], INbreast [46], and MIAS (http://peipa.essex.ac.uk/info/mias.html (accessed on 10 October 2019)). For evaluation of the proposed framework, a 50:50 approach has opted which means 50% of the images of each dataset are consumed for the training and remaining for testing. A few sample images of each dataset are illustrated in the figures. The each dataset description is given below.

**Figure 2:**
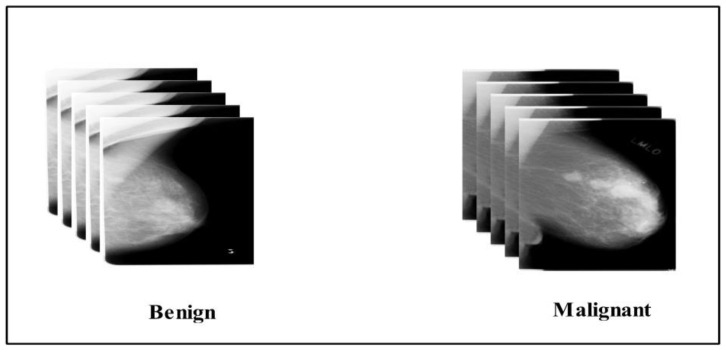
Sample images CBIS-DDSM.

CBIS-DDSM: The Curated Breast Imaging Subset of Digital Database for Screening Mammography (CBIS-DDSM) is an improved and standardized form of DDSM. A trained mammographer curated the dataset. The images are in the Dicom form. The ROI annotations are also provided of the images. The two views craniocaudal(CC) and mediolateral oblique (MLO) are available. The 1696 mass images with pathological information training and testing are available [45]. Figure 2 shows a few examples of images from this dataset.

**Figure 3:**
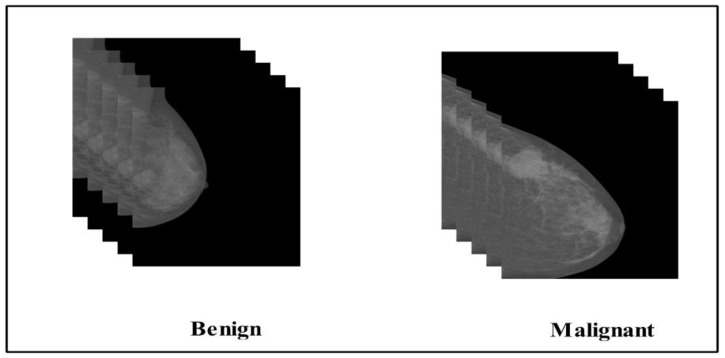
Samples Images of INbreast dataset.

**Figure 4:**
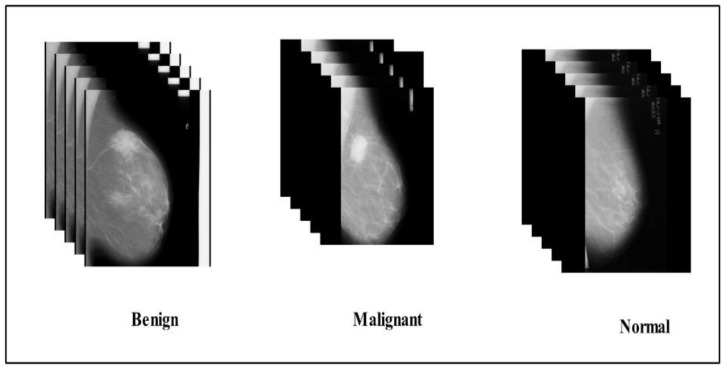
Sample images of MIAS dataset.

INbreast: The Portugal breast research group generated INbreast dataset. The INbreast database includes 410 images from 115 patients. The 108 mass mammogram images with BIRADS information are available. The 107 images with mass annotations are available. The INbreast images are available in Dicom format. The images size is 3328 × 4084 or 2560 × 3328 pixels [46]. The 108 mass mammogram images are taken for the experiment. Figure 3 represents a few sample images as well.

mini_MIAS (Mammographic Image Analysis Society): This is publicly available dataset. There are 322 images in this dataset. MIAS images have been condensed to 200-micron pixel edges. Every image is 1024 × 1024 pixels. The benign, malignant, and normal images are given. The complete information of the dataset regarding normal, benign, and malignant images is available (http://peipa.essex.ac.uk/info/mias.html (accessed on 10 October 2019)). The images are available in a portable gray map (PGM). The 300 images without calcification cases are taken for the experiment. Figure 4, represent sample images of this dataset.

These three datasets CBIS-DDSM, INbreast, and mini-MIAS are converted into portable network graphic (PNG) format [47]. The resizing of the images is performed by using the neighbor interpolation method into 256×256.

### 2.2. Data Augmentation

To enable the deep learning models the sample images increased by using data augmentation [47]. The deep learning models give promising results on a large amount of data. In this work, three mathematical operations are implemented such as flip left to right, flip up to down, and rotation at 90 degrees. Algorithm 1 of data augmentation is presented below.

**Algorithm 1:** Data AugmentationWhile (i = 1 to target object) Step 1: Input read Step 2: Flip Left to right Step 3: Flip-up to down Step 4: Rotate image to 90°
Step 5: Image write step 2 Step 6: Image write step 3 Step 7: Image write Step 4 End

**Figure 5:**
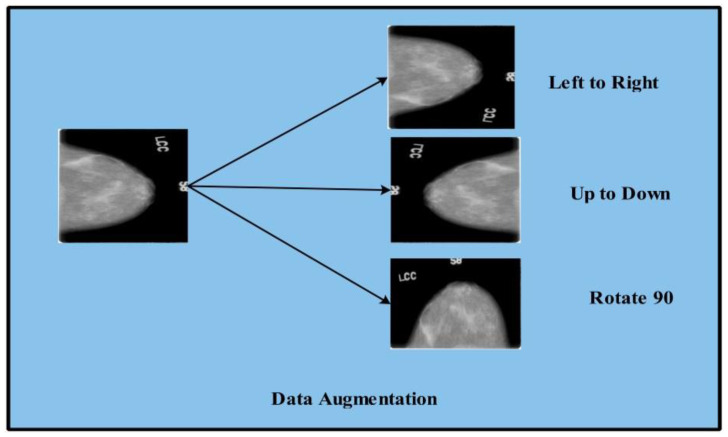
Data augmentation.

In Figure 5, data augmentation is presented of the CBIS-DDSM [45] images. The augmentation of the data is performed by using flip left to right, up to down, and by rotating at 90°.

Table 1 shows the detailed information of the three datasets CBIS-DDSM, INbreast, and MIAS. The detail of original images and data augmentation is given below.

### 2.3. Convolutional Neural Network

There are several layers in the CNN model, including an input layer, convolutional layers, batch normalization layers, Pooling, ReLu, softmax layers, and one output layer. The input layer consist of dimensions a×b×c of the input image. The number of channels described by c. The convolutional layer that is main and first layer utilizes three inputs: a, b, and c. The mapping of features is performed in the convolutional layer. These features are utilized for visualization and used in the activation layer.

### 2.4. Fine-Tuned MobilenetV2

The MobilenetV2 model is a portable custom-based model in computer vision. This model sustains the same accuracy by decreasing the number of operations and consuming a small amount of memory. In this model, the inverted residual layer with a linear bottleneck is included. In this model, the compressed representation of low-dimensional input is used that is converted into high dimension by using the light weight depth-wise convolution filters [48].

MobilenetV2 performs efficiently in any framework. This model reduces the need for main memory in many embedded hardware designs while providing a small amount of cache memory that increases the speed and efficiency of the system. It reduces the main memory need. The MobilenetV2 performs best in object detection, semantic segmentation, and classification tasks. In Mobilenetv2, depth-wise convolution, linear bottleneck, inverted residuals, and information flow interpretation are used [49].

The depth-wise separable convolution blocks achieve good performance. In MobilenetV2, the convolution layers are replaced with the two other layers. The depth-wise convolution that is the first layer uses the single convolution filter per input unit to perform the lightweight filtering. The pointwise convolution that is the second layer generates new features by utilizing the input channels of the linear combinations. In the residual bottleneck, the information in the deep convolutional layer is encoded in some manifold that is residing in a low-dimensional subspace. This procedure can be captured by reducing the layer dimensionality and operating space dimensionality.

The manifold expands the space by allowing us to reduce the activation space dimensionality. The deep convolution neural network has ReLU, which is a nonlinear per coordinate transformation that breaks down intuition. If the volume of the manifold of interest after ReLU transformation remains non-zero, the linear transformation is formed. ReLU has complete information about the input manifold if it remains in the low-dimensional subspace of the input space. The inverted residuals are built up that is more memory efficient [49].

In fine-tuned MobilenetV2 the last three layers replaced by adding new layers according to the target datasets. The target dataset is based on mini-MIAS, CBIS-DDSM, and INbreast. To train the fine-tuned model transfer learning approach is used. In the training process, 100 epochs, 0.00001 learning rate, and 8 batch size is set. The Single Shot Multibox Detector (SSD) [50] and Adam optimizer are utilized for the learning method. To quantize bounding box space, the SSD uses default anchor boxes with different fractions and measures. SSD adds different feature layers in the network end [2]. Finally, deep features are extracted for further processing from the fine-tuned model of layer global average pool (GAP). The vector output size of this layer is N×1280.

### 2.5. Fine-Tuned Nasnet Mobile

The Nasnet Mobile is a search network of neural architecture. By using a small dataset, the architectural building block searched and transferred on the large dataset. The best cells or convolutional layers are searched and applied to the Imagenet by making more copies of the convolution layers. The new method ScheduleDropPath regularization is proposed that improves the generalization of the Nasnet models [51]. The samples child of the different networks is added in the recurrent neural network (RNN) to propose the NAS [52]. To achieve accuracy the network child is trained. To update the controller, the resulting accuracies are used that generate the best architecture. The controller weights are updated by using the gradient. The RNN controller only returns the structure of the normal and reduction cells [51]. The Nasnet search space is familiar with CNN architecture engineering because it identifies motifs such as convolutional filter bank combinations, nonlinearities, and connections prudent selection [53,54,55].

The above-mentioned studies suggests to predict the generic convolutional cells that are utilized to express motifs for the controller RNN. To control the filter depth and spatial dimensions of the input the cells are stacked in the form of series. While in Nasnet convolutional, nets manually pertain to the architecture. These are built up by convolutional cells and repeated several times by using different weights but the same architecture [51].

In Nasnet proposed by [51], the reinforcement learning search method is used to search the blocks. The initial convolutional filters and motif repetitions N is free from the parameters and used in scaling. The features map of the same dimensions is returned by the convolutional cells when in normal cell form, otherwise in reduction cells the features map with their height and width reduced by a factor of two.

The Nasnet model uses scalable, convolutional cells from data and can be transferred to the other image classification tasks. The parameters and computational cost of the architecture are quite flexible and this model can be used model for a lot of different problems. The search space is used to minimize the architecture complexity from the depth of the network. The searching space achieves good architecture on small datasets and shifts the learned architecture to the classification.

The last three layers of the Nasnet are replaced by adding new layers based on the target dadasset during the fine-tuning phase. The target dataset consists of mini-MIAS, CBIS-DDSM, and INbreast. The transfer learning approach is used to train the fine-tuned models. The number of epochs used to train the process is 100, the learning rate is 0.00001, and the batch size is 8. To learn the methods, the Adam optimizer and SSD are used [50]. To quantize bounding box space, the SSD uses default anchor boxes with different fractions and measures. SSD adds different feature layers in the network end [2]. Finally, deep features are extracted for further processing from the fine-tuned model of the layer Global Average Pool (GAP). This layer’s output vector size is N*1056.

### 2.6. Transfer Learning

Transfer learning makes use of an already trained and reused model as the foundation for a new task and model. The model used for one task can be repurposed for other tasks as an optimization to improve performance. By applying transfer learnin the model can be train with a small volume of data. It is helpful to save time and achieve good results [56,57].

In the transfer learning approach, we transfer knowledge from the source mammogram input images $I_{s}$ to the target domain mammogram mass images $I_{T}$. The target classifier $T_{c}\;(M_{t})$ is to be trained from the input mammogram image $I_{s}$ to the target image $I_{T}$ to get the classifier prediction about $BMN_{Ti}$, which stands for benign, malignant, and normal. To extract the features transfer layer is used. The top layer from the classifier retrained the new target classes while the other layers kept frozen.

$$BMN_{Ti\;}=T_{c}\;(M_{t})$$

**Figure 6:**
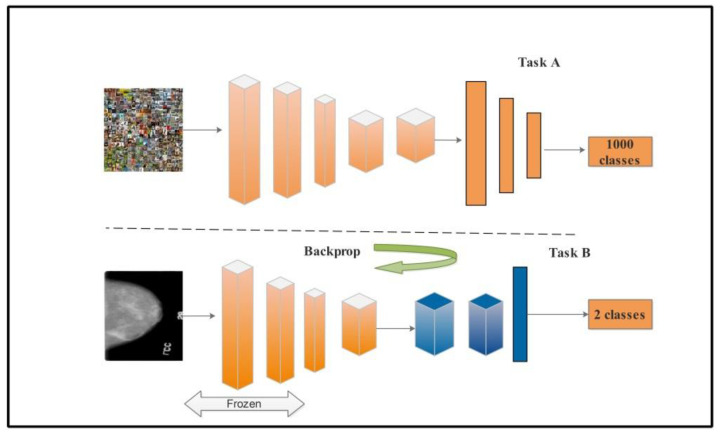
Transfer learning architecture.

To extract the features from MobilenetV2 and Nasnet the transfer learning approach is used. In Figure 6, multiple classes of knowledge have been utilized into two classes.

### 2.7. Whale Optimization Algorithm (WOA)

To explore the feasible solution to the problems in the search space, whale individuals are used in the community. There are three functions performed by WOA: encircling, shrinking, and hunting. In the exploitation phase, the encircling and shrinking operations are used, while in the exploration phase, the hunting function is used [58].

To provide the solution of the dimension optimization problems (DO) the procedures of the ith individual in the cth generation is used to find the best solution. 

The WOA procedures are following.

Encircling Operation

(1) $$ESH_{ij}\left(c+1\right)=ESH_{*j\;}\left(c\right)-B\cdot O_{ij}\left(c\right)$$

Shrinking Operation

(2) $$ESH_{ij}\left(c+1\right)=ESH_{*j}\left(c\right)+g^{et}\;\cdot \cos\left(2\pi t\right)\cdot {O^{\prime }}_{ij}\left(c\right)$$

Hunting Operation

(3) $$ESH_{ij\;}\left(c+1\right)=ESH_{kj\;}\left(c\right)-B\cdot O_{ij}^{*}\;\left(c\right)$$

(4) $$B=2\left(1-\frac{c}{c_{max}}\right)\cdot \left(2rd-1\right)$$

The arbitrary number in the range [0 1] is described by (rd), The present number of the iterations is represented by *c*, iterations maximum no is described by $c_{max}$, the best solution positive vector is represented by $ESH_{*}\left(c\right)$. To define logarithmical spiral shape the constant e is used, and the random number in [−1, 1] is represented by  t. The arbitraryposition vector $ESH_{K}\left(c\right)$ is selected from the present population. Three distances are following. The first is $\left|O_{ij}\;\left(c\right)=\right|2rd.ESH_{*j}\;\left(c\right)-ESH_{ij}\left(c\right)|$, the 2nd distance is ${O^{\prime }}_{ij}\;\left(c\right)=\left|ESH_{*j}\;\left(c\right)-ESH_{ij\;}\left(c\right)\right|$, and the 3rd distance is $O^{*}{}_{ij}\left(c\right)=\left|2rd.ESH_{kj}\left(c\right)-ESH_{ij}\left(c\right)\right|$. According to the probability $p_{rob}$, Equations (1)–(3) are executed by WOA. The whale individuals are updated by Equation (1), when $p_{rob}$ < 0.5 and |B| < 1; otherwise individuals are corrected by Equation (3), when |B| ≥ 1. The Equation (2) is used to update the individuals, when $p_{rob}$ ≥ 0.5. 

#### 2.7.1. Modified Entropy Whale Optimization Algorithm (MEWOA)

The WOA learns the best current solution from the exploitation phase, which easily succumbs to local optimization and reduces population diversity. The random individual learning operation has some sightlessness and does not perform any effective interchange of information between groups in the exploration phase, which disrupts the algorithm convergence rate. The WOA needed to be improved to reduce these issues. The new algorithm MEWOA is proposed. To balance the WOA’s an exploration and exploitation functions the control parameter B is used.The exploration probability in WOA is only 0.1535, during the iterative process of the algorithm.WOA has limited ability. The development and exploration process in the MEWOA is controlled by linearly increasing the probability. Individual quality in the animal large group improves when individuals learn from the elite and other members of the group. Individual neighborhoods are formed through adaptive social learning procedures that use the social position of the individual, social influence, and social network formation. The adaptive social networking approach is used to build whales’ adaptive community and to improve the interaction between groups, as well as to improve the MEWOA’s calculation accuracy. The new approach is proposed based on neighborhood, which will also increase the population diversity. The MEWOA’s convergence speed increases when the population jumps out from a local optimum by introducing the wavelet mutation strategy, and the algorithm exhibits premature convergence when the population falls into the local optimum [58].

#### 2.7.2. Linear Increasing Probability

The control parameters |B|∈[0, 2] in the WOA, the global exploration is performed by the algorithm when |B| ≥1. As presented in Equation (4), when $c\geq \frac{1}{2}\;c_{max},\;\left|B\right|<1$ is always true. The algorithm has weak exploration ability in the second half of the iteration. Let $q=2\left(1-\frac{c}{c_{max}}\right)$, ᴧ = 2rd − 1, then B = q.ᴧ in the whole iteration, and the probability of |B≥1| is

(5) $$P_{rob}\left(\left|B\right|\geq 1\right)=0+\int_{1}^{\;2}\int_{1_{/q}}^{1}O\Lambda Oq\;\approx 0.307.$$

The WOA performs exploitation operations when the $p_{rob}\;\geq 0.5$ and the exploration probability is 0.5 × 0.307 = 0.1535 in the iterations. The search ability of the MEWOA is not maintained by |B| due to weak exploration ability, so the exploitation and exploration ability is handled by probability $P_{i}$ that will increase the number of iterations linearly to conduct global exploration.

(6) $$P_{i}=0.5+x.\frac{c}{c_{max}}$$

where 0.2≤x<0.5.

The $r_{no}$ is arbitrary no in [0 1]. The exploitation operation is performed when the $r_{no\;}<p_{i}$; otherwise, an exploration operation is performed by the algorithm. The global exploration has a possibility of 0.1 when the coefficient of $\frac{C}{c_{max}}<0.5$ even in the last iteration, which will rise algorithm capacity to jump out of local optimization.

Average exploration probability according to Equation (6) will be

(7) $$\bar{P_{i}}=1-\frac{1}{c_{max}}\;.\;\overset{c_{max}}{\underset{c=1}{\sum }}\left(0.5+0.4.\frac{c}{c_{max}}\;\right)=0.3{-}_{\frac{0.2}{c_{max}}}$$

when $c_{max}\geq 2,\;\overset{ˇ}{P_{i}}\geq 0.2>0.1535$. The exploitation and exploration is controlled by linear increasing probability $P_{i}$ that will increase the algorithm search ability.

#### 2.7.3. Adaptive Social Learning Strategy

In social behavior, each whale can build a neighborhood membership relationship and can change its current best solution behavior of imitation. The algorithm (MEWOA) moves away from the local optimal solution by improving and enhancing information sharing between groups. For the current population, $G\left(c\right)=\left\{ESH_{1}\left(c\right),ESH_{2}\left(c\right),\ldots \ldots .ESH_{PN}\left(c\right)\right\}$, the population size is denoted by *PN*. The fitness value is computed and ordered from minor to huge to achieve the stored population $G_{1}\;\left(c\right)=\{ESH_{\left(1\right)}\left(c\right),\;ESH_{\left(2\right)}\left(c\right),\ldots \ldots .,ESH_{\left(PN\right)}\left(c\right)$}, and $ESH_{\left(i\right)}$(c) is used to describe the social ranking.

(8) $$SR_{\left(i\right)}\left(c\right)=PN+1-i,\;i=1,2,\ldots \ldots \ldots PN.$$

$ESH_{i}\left(c\right)$ social impact is

(9) $$t_{i}\left(c\right)=\frac{SR_{\left(i\right)}\left(c\right)*S_{if\;}}{PN},\;i=1,2,\ldots \ldots \ldots \ldots PN$$

The social impact is represented by $S_{if}$, and $S_{if}\leq 0.4$. Equations (8) and (9) defined that when the social impact is greater the social ranking is also greater that denote the better individual and by using the specific limit of $S_{if}$ the influence will be limited. For $G_{1}\left(c\right)$ population, the social network is constructed according to social influence. The relationship between $ESH_{\;i}\left(c\right)$ and $ESH_{j}\left(c\right)$ is defined as

(10) $$SR_{\left(ij\right)}\left(c\right)=\{\begin{array}{c} 1,\;if\;\;\;\;\;\;\;rd_{1}\leq max\left(t_{i}\left(c\right),t_{j}\left(c\right)\right)\\ 0,\;otherwise \end{array}$$

where $rd_{1}$ is a random number in [0, 1]. When the social influence is greater, the individual has the strongest connection with other individuals as shown in Equation (10), and enhance the likelihood possibility ($t_{\left(j\right)}\left(c\right))$, and when there is less social influence, the likelihood $\left(t_{i}\left(c\right)\right)$ of the relationship enhances between the individuals and other individuals. More Individuals can adopt the best individual behavior. The greater an individual’s social influence, the more interaction between the individuals. The $ESH_{i}\left(c\right)$ the adaptive neighborhood of individuals built up the relationship between individuals:

(11) $$PN_{\left(i\right)}\left(c\right)=[ESH_{\left(j\right)}\left(c\right)|j\in \left[1,\;PN\right]\;\;\;\;and\;\;\;\;J\neq i\;\;\;\;and\;\;\;\;\;SR_{\left(ij\right)}\left(c\right)=1].$$

In the algorithm, the exploitation stage is in the center of the best search solution, and the exploration ability is finished due to interaction between the group members. The new search strategy of a whale is recognized using community adaptive strategy and linearly increasing probability. The new strategy is described here.

If $p_{rob1\;}<p_{i}$, the jth dimension of ith individual, $ESH_{\left(i\right)}\left(c\right)$ in population $G_{1}\left(c\right)$ updates its position as follows.

(12) $$ESH_{\left(i\right)j}\left(c+1\right)=\{\begin{array}{c} ESH_{\left(1\right)j}\left(c\right)-B.O_{\left(i\right)j}\left(c\right)\;\;\;\;\;\;\;\;rd^{\prime }<0.5\\ ESH_{\left(1\right)j}\left(c\right)+g^{et}\;.\cos\left(2\pi t\right).\;{O^{\prime }}_{\left(i\right)j}\;\left(c\right)\;\;\;\;\;\;\;rd^{\prime }\geq 0.5 \end{array}$$

where $O_{\left(i\right)j}\left(c\right)=\left|2rd.ESH_{\left(1\right)j}\left(c\right)-ESH_{\left(i\right)j}\left(c\right)\right|,\;{O^{\prime }}_{\left(i\right)j}\left(c\right)=\left|ESH_{\left(1\right)j}\;\left(c\right)-ESH_{\left(i\right)j}\left(c\right)\right|$. if $prob_{1}\geq \;p_{i}$, the adaptive neighborhood procedure is used by the algorithm to explore. This process is described in Equation (13). Let the following:

$$f_{\left(i\right)1}=B.\overset{U_{i}}{\underset{U=1,ESHm_{U\;}\left(c\right)\in PN_{i}\left(c\right)}{\sum }}W_{U}.(2rd.ESH_{\left(i\right)j}\left(c\right)-ESHm_{U^{J}}\left(c\right))$$

$$f_{\left(i\right)2}=g^{et}.\cos\left(2\pi t\right).\overset{U_{i}}{\underset{U=1,{\mathrm{ESHm}}_{U}\left(c\right)\in {\mathrm{PN}}_{\left(i\right)}\left(c\right)}{\sum }}\;W_{U}\left(ESH_{\left(i\right)j}\left(c\right)-ESHm_{U}j\left(c\right)\right)$$

Then,

(13) $$ESH_{\left(i\right)j}\left(c+1\right)=\{\begin{array}{c} ESH_{\left(1\right)j}\left(c\right)-f_{\left(i\right)1}\;\;\;\;rd^{\prime }<0.5\\ ESH_{\left(1\right)j}\left(c\right)+f_{\left(i\right)2}\;rd^{\prime }\geq 0.5 \end{array}\;\;{}_{Prob_{2}<0.5}$$

where $Prob_{1},prob_{2},rd\;and\;rd^{\prime }$ are the arbitrary number in [0, 1], $P_{i}$ is represented in Equation (6). $U_{i}$ is the cardinality of $PN_{\left(i\right)}$(c), $U_{i}$= |$PN_{\left(i\right)}\left(c\right)|$. $W_{U}$ is the weight, $W_{U}=\frac{SRm_{U}\;\left(c\right)}{\sum_{U=1}^{U_{i}}SRm_{U\;}\left(c\right)}$. Using Equations (12) and (13), updating individuals fully utilizes the most recent best solution and individual adaptive neighborhood information while effectively increasing population diversity.

#### 2.7.4. Morlet Wavelet Mutation

MEWOA holds the key to breaking out of the local optimum for optimization problems involving extreme points of intensive distribution. In biological growth, change is the main factor. To adjust the mutation space dynamically that increases the solution frequency. The amplitude function can be reduced by fixing the wavelet function, extending parameters, and fixing the mutation space of the number of iterations to a specific limit, the change operation can be grasped by using the fine-tuning effect. The WOA is incorporated by using the wavelet mutation to improve the algorithm’s convergence and correctness speed and by allowing it to release from local optimization by enhancing its ability. The purpose of the change in the algorithm’s exploration phase is to find the best solution from all other solutions.

Suppose the $Prob_{m}$ is the mutation probability, and random no in [0, 1] is represented by rd. When $P_{rob1}\geq P_{i}$ and $\;rd\geq prob_{m}$, modified wavelet mutation represent the position of whale according to $prob_{m}$.

$$f_{\left(i\right)3}=\sigma j.\left(y_{j}-ESH_{\left(i\right)j}\left(c\right)\right)$$

$$f_{\left(i\right)4}=\sigma j.\left(ESH_{\left(i\right)j}\left(c\right)-t_{j}\right)$$

(14) $$ESH_{\left(i\right)j}\left(c+1\right)=\{\begin{array}{c} ESH_{\left(i\right)j\left(c\right)+f_{\left(i\right)3}}\;\;\;rd^{\prime }<0.5\;\\ ESH_{\left(i\right)j\left(c\right)+f_{\left(i\right)4}}\;\;\;\;\;rd^{\prime }\geq 0.5 \end{array}\;\;\;\;P_{rob2}\geq 0.5$$

The random number is represented by $P_{rob1}$, $P_{rob2}$, rd, and rd′ in the range of [0 1] as mentioned above. The upper and lower bounds of jth dimensions are described by $y_{j}$ and $t_{j}$ The  σ coefficient wavelet mutation is $\sigma_{j}=\frac{1}{\sqrt{v}}\;\$\left(\frac{\emptyset J}{v}\;\right),\;J\;\in \left\{1,2,\ldots .,DO\right\}$. The Morlet Wavelet mutation is $(ESH) and $\$\left(ESH\right)=g-\frac{esh^{2}}{2}\;.\cos\left(5\mathrm{ESH}\right)$, the function energy 99% is consists of [−2.5, 2.5], so ∅J in [−2.5v, 2.5v] is represented as a random number.

When iterations increase the scaling parameter v also increases makes possible for the algorithm to find the best solution when there are huge at the end of the iteration.

(15) $$v=a\left(\frac{1}{a}\right)\left(1-\frac{c}{c_{max}}\right)$$

The constant number is represented by a. The proposed Algorithm 2 MEWOA is presented below.

**Algorithm 2:** Modified Entropy Whale Optimization Algorithm.
Start Parameters to initialize MEWOA such as a, $\;c_{max}$, e, t, PN, $S_{if}$,$prob_{m}$ The initial population randomly generates G$\left(0\right)=\{ESH_{1}\;\left(0\right),ESH_{2}\left(0\right),\ldots \ldots \ldots \ldots \ldots \ldots \ldots ,ESH_{PN}\left(0\right)\}$ Assess each whale’s individual fitness values, $ESH_{*}\left(0\right)$, search individual best c = 1 While (**$c<c_{max})$** Update $p_{i}$ according to Equation (6), compute the neighborhood $PN_{\left(i\right)\left(c\right)}$ of whale, for each search individual $ESH_{\left(i\right)\left(c\right)}$ according to Equations (8)–(11) If ($Prob_{1}<p_{i})$  update the whale individual by using Equation (12); Else If $Prob_{2}<0.5$ Use Equation (13); Else Use Equation (14); End if End if End for Fix boundaries of the whale individuals that go beyond. Evaluate individual whale fitness values; Update the $ESH_{*}\left(c\right)$ global best solution. c=c+1; End while Output the best search individual $ESH_{*}$; End

## 3. Results

The experimental results are offered in this section by using three datasets: CBIS-DDSM, Mini-MIAS, and INbreast. The detail of the datasets is given in Section 2.1. The results of each dataset are measured by applying the deep learning models from a different perspective. For the validation purpose, several classifiers of machine learning are applied by using 10-fold cross-validation. In the 10-fold cross-validation test, the provided learning set is divided into ten distinct subsets of comparable size. 

The number of subsets created is referred to as the fold in this context. Then, these subsets are used for training and testing, and the loop is repeated until the model has trained and tested every subset, whereas the 10-fold cross-validation performed better than any other k fold selection. 

As a result, the 10-fold cross-validation method is used to validate the models in order to avoid over- and under-fitting during the training process. Different measures like Sensitivity, Precision, F1-Score, AUC, FPR, Accuracy, and Time are computed to evaluate the performance of the proposed method. All the training is conducted on MATLAB2020a by using a Personal Computer of 16 GB Ram and a 4 GB graphics card.

### 3.1. Experimental Results

1. Several experiments are conducted to validate the proposed method.
2. Classification using Fine-tuned MobilenetV2 deep features.
3. Classification using Fine-tuned Nasnet Mobile deep features.
4. Classification using MEWOA on Fine-tuned MobilenetV2 deep features.
5. Classification using MEWOA on Fine-tuned Nasnet Mobile deep features.
6. Classification using serial-based non-redundant fusion approach.
7. Classification using MEWOA on fused features.

### 3.2. Classification Results

**Figure 7:**
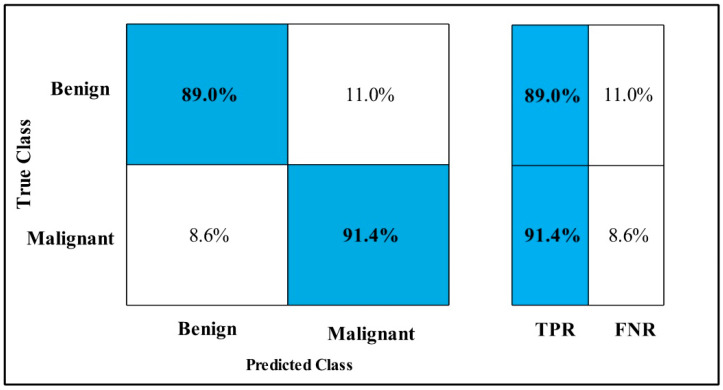
Fine-tuned MobilenetV2 TPR CBIS-DDSM.

The classification results are conducted on three datasets. Several classifiers are applied to compute the classification results. In Table 2, the Fine-tuned MobilenetV2 model is applied to the CBIS-DDSM dataset. The deep features of the dataset are extracted from the GAP layer and fed to classifiers. The highest accuracy is 90.3%, which is achieved by the Cubic SVM classifier in 260.13 (s). The minimum time is taken by Gaussian Naïve Bayes is 20.432 (s), but the accuracy is 71.5%, which is less than that of Cubic SVM. The second highest accuracy is achieved by Weighted KNN, which is 88.2% in 72.92 (s). The sensitivity rate of each classifier is also calculated, and the best-noted value is 90.20% for Cubic SVM. The sensitivity can be confirmed by using a confusion matrix, as mentioned in Figure 7. The machine learning classifiers Cubic SVM, Fine Tree, Linear SVM(LSVM), Quadratic SVM(QSVM), Fine Gaussian SVM (FG-SVM), Gaussian Naïve Bayes (GN-Bayes), Fine KNN (FKNN), Medium KNN (MKNN), Weighted KNN (WKNN), and Coarse KNN(Co-KNN) are applied to classify the mammography images.

In Table 3, the Fine-tuned Nasnet model is applied to the CBIS-DDSM dataset. The deep features of the dataset are extracted from the GAP layer and fed to classifiers. The highest accuracy is 93.9%, which is achieved by the Cubic SVM classifier in 112.96 (s). In this table, the minimum time is taken by Fine Tree, which is 16.91 (s), but the accuracy is 89.8%, which is less than Cubic SVM. 

**Figure 8:**
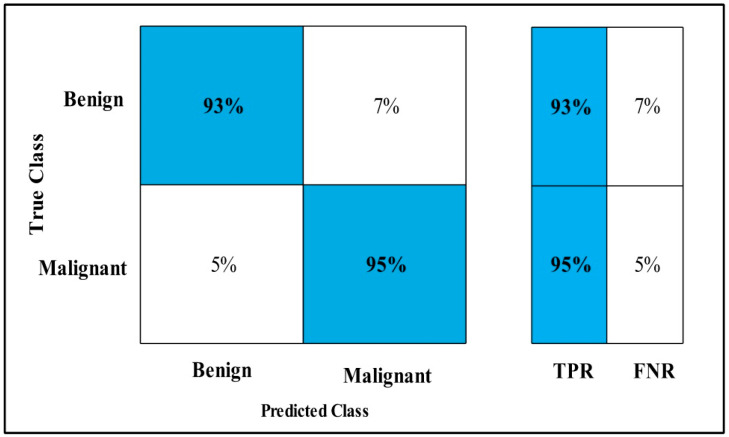
Fine-tuned Nasnet CBIS-DDSM.

The second highest accuracy is achieved by WKNN, which is 93.6% in 59.909 (s). Each classifier sensitivity rate is also computed, and the Cubic SVM achieved the best-noted value that is 94%. A confusion matrix, as shown in Figure 8, can be used to confirm it.

In Table 4, MEWOA on MobilenetV2 is applied to the CBIS-DDSM dataset. The deep features of the dataset are extracted from the GAP layer and fed to classifiers. The highest accuracy is 90.0%, which is achieved by Cubic SVM in 132.98 (s). In this table, the minimum time is taken by GN-Bayes, which is 8.70 (s), but the accuracy is 70.5%, which is less than Cubic SVM. 

**Figure 9:**
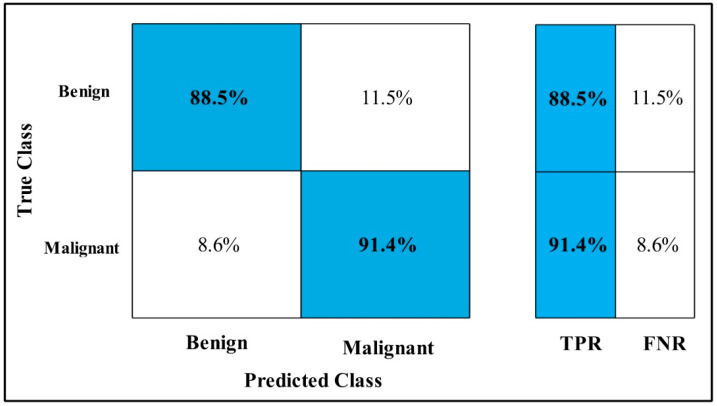
MEWOA TPR on MobilenetV2 CBIS-DDSM.

The second highest accuracy is achieved by WKNN, which is 87.4% in 37.385 (s). Every classifier sensitivity rate is also calculated, and Cubic SVM achieved the best-noted value that is 89.95%. A confusion matrix, as shown in Figure 9, can be used to confirm it.

In Table 5, MEWOA on Nasnet is applied to the CBIS-DDSM dataset. The deep features of the dataset are extracted from the GAP layer and fed to classifiers. The highest accuracy is 93.50%, which is achieved by Cubic SVM in 73.24 (s). 

**Figure 10:**
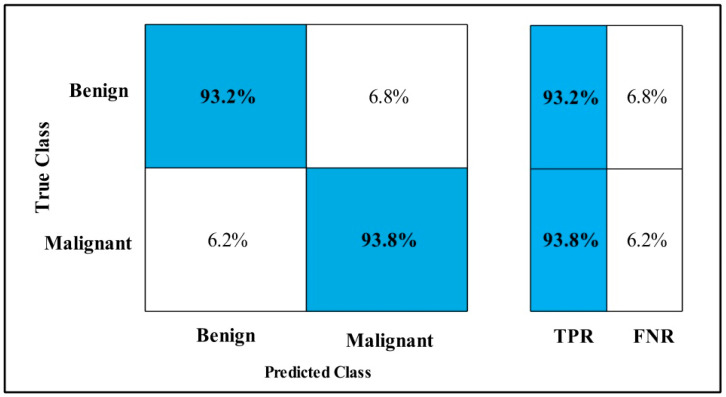
MEWOA TPR on Nasnet Mobile CBIS-DDSM.

In this table, the minimum time is taken by GN-Bayes, which is 11.57 (s), but the accuracy is 83.7%, which is less than Cubic SVM. The second highest accuracy is achieved by QSVM, which is 92.30% in 75.26 (s). The sensitivity rate of each classifier is also computed, and the Cubic SVM achieves the best-noted value that is 93.50%. The sensitivity can be calculated by the confusion matrix, as illustrated in Figure 10.

In Table 6, by using CBIS-DDSM dataset, Serial Fusion on MobilenetV2 and Nasnet deep features is applied. The GAP layer extracts deep features from the dataset and feeds them to classifiers.

**Figure 11:**
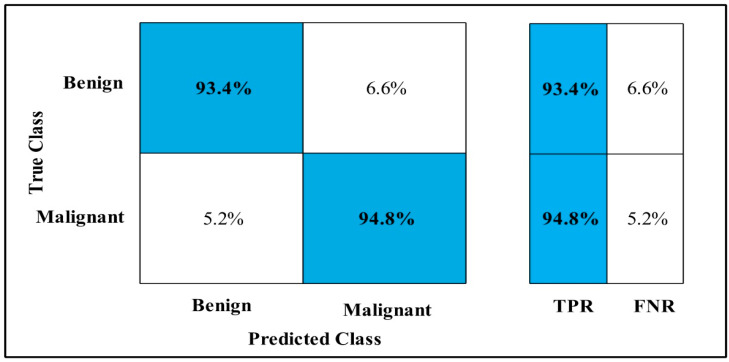
Fusion on MobilenetV2 and Nasnet TPR for CBIS-DDSM.

The highest accuracy is 94.1%, which is achieved by Cubic SVM in 314.97 (s). In this table, the minimum time is taken by GN-Bayes, which is 55.161 (s), but the accuracy is 85.5%, which is less than Cubic SVM. The second highest accuracy is achieved by QSVM, which is 93.0% in 265.45 (s). Each classifier sensitivity rate is also calculated and the Cubic SVM has the best-noted value that is 94.1% and can be confirmed by using the confusion matrix as described in Figure 11.

In Table 7, MEWOA on fusion is applied to the CBIS-DDSM dataset. The deep features of the dataset are extracted from the GAP layer and fed to classifiers. 

The highest accuracy is 93.8%, which is achieved by Cubic SVM in 255.84 (s). In this table, the minimum time is taken by Fine Tree, which is 42.42 (s), but the accuracy is 88%, which is less than Cubic SVM. 

**Figure 12:**
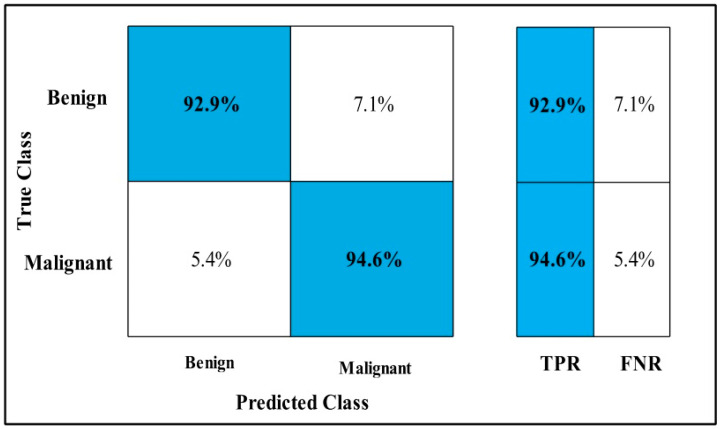
MEWOA on fusion TPR for CBIS-DDSM.

The second highest accuracy is achieved by QSVM, which is 93.0% in 227.28 (s). Each classifier sensitivity rate is also calculated and Cubic SVM has a best-noted value that is 93.75% and can be verified by using the confusion matrix as defined in Figure 12.

**Figure 13:**
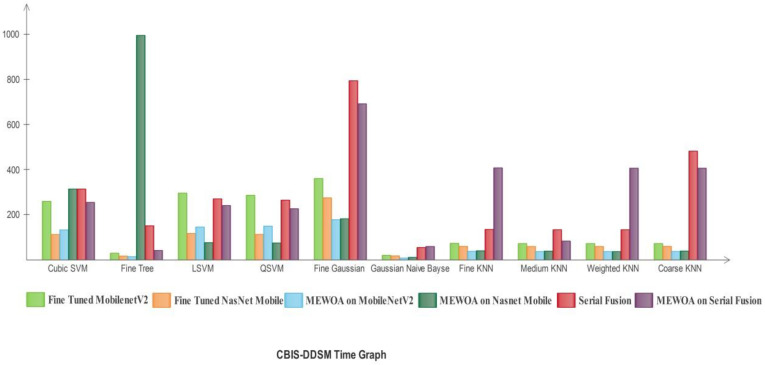
Time comparison with individual machine learning classifiers of deep learning models for CBIS-DDSM.

In Figure 13, the deep learning model’s time comparison graph by using the machine learning classifiers is shown. 

The Fine Tree utilized maximum time in the MEWOA in Fine-tuned Nasnet model. The FG-SVM classifier utilized maximum time in serial fusion. The GN-Bayes utilized minimum time.

In Table 8, the Fine-tuned MobilenetV2 model is applied to the MIAS dataset. The GAP layer is used to extract the deep features and fed to classifiers. 

The highest accuracy is 99.4%, which is achieved by the Cubic SVM classifier in 85.29 (s). In this table, the minimum time is taken by Fine Tree, which is 22.82 (s), but the accuracy is 88.9%, which is less than Cubic SVM. 

**Figure 14:**
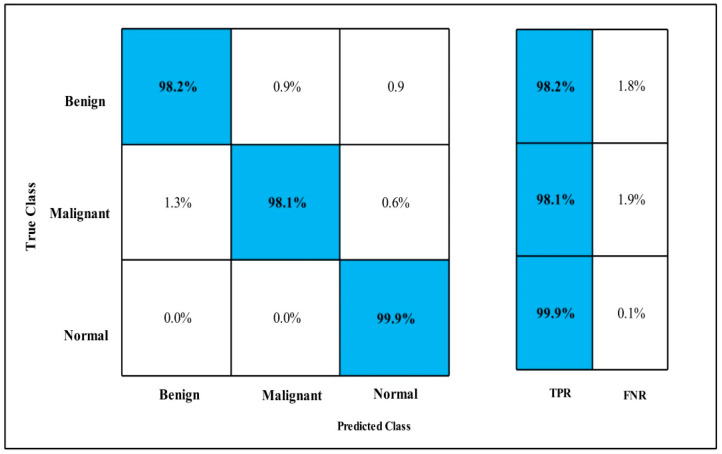
Fine-tuned MobilenetV2 TPR for MIAS.

The second highest accuracy is achieved by QSVM, which is 99.3% in 79.88 (s). Each classifiers sensitivity is computed and Cubic SVM has 98.73% best-noted value that can be verified by using the confusion matrix that is presented in Figure 14.

In Table 9, the Fine-tuned Nasnet model is applied to the MIAS dataset. The GAP layer extracts the dataset’s deep features and feeds them to the classifiers. 

The highest accuracy is 99.7%, which is achieved by the WKNN classifier in 81.459 (s). In this table, the minimum time is taken by Fine Tree, which is 35.187 (s), but the accuracy is 99.1%, which is less than WKNN. 

**Figure 15:**
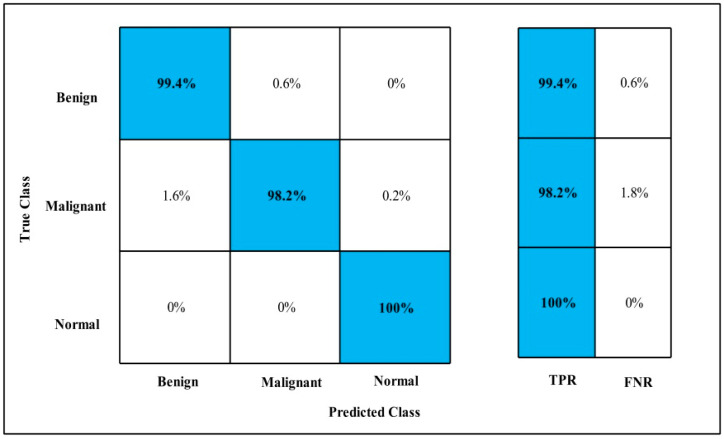
Fine-tuned Nasnet TPR for MIAS.

The second highest accuracy is achieved by Cubic SVM, which is 99.6% in 267.43 (s). Every classifier sensitivity rate is computed and WKNN has a 99.2% best sensitivity rate that can be confirmed by using a confusion matrix, as described in Figure 15.

In Table 10, MEWOA on Fine-tuned MobilenetV2 model is applied to the MIAS dataset. The GAP layer is used to extract the deep features and fed to classifiers. 

The highest accuracy is 99.4%, which is achieved by the Cubic SVM classifier in 75.49 (s). In this table, the minimum time is taken by Fine Tree that is 20.09 (s), but the accuracy is 89.1%, which is less than Cubic SVM. 

**Figure 16:**
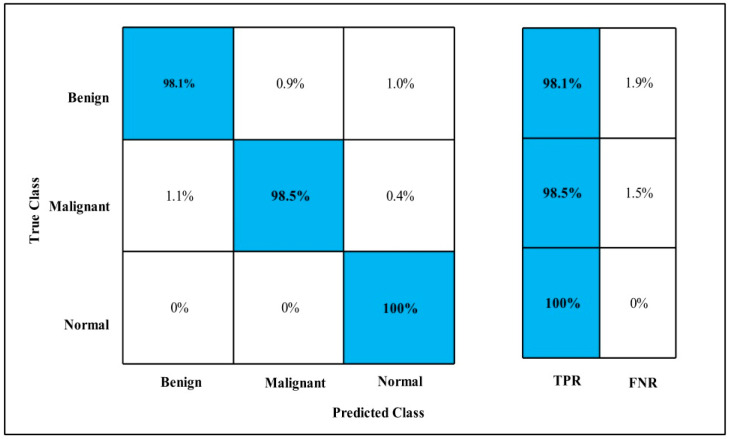
MEWOA on Fine-tuned MobilenetV2 TPR for MIAS.

The second highest accuracy is achieved by FKNN, which is 99.3% in 65.81 (s). Each classifier sensitivity rate is calculated and Cubic SVM has a best-noted value that is 98.87%. The sensitivity rate can be confirmed by using the confusion matrix as described below in Figure 16.

In Table 11, MEWOA on the Fine-tuned Nasnet model is applied to the MIAS dataset. The deep features are extracted from the GAP layer and fed to classifiers. The highest accuracy is 99.7%, which is achieved by the WKNN classifier in 24.70 (s). In this table, the minimum time is taken by Fine Tree, which is 9.40 (s), but the accuracy is 98.9%, which is less than that of WKNN. 

**Figure 17:**
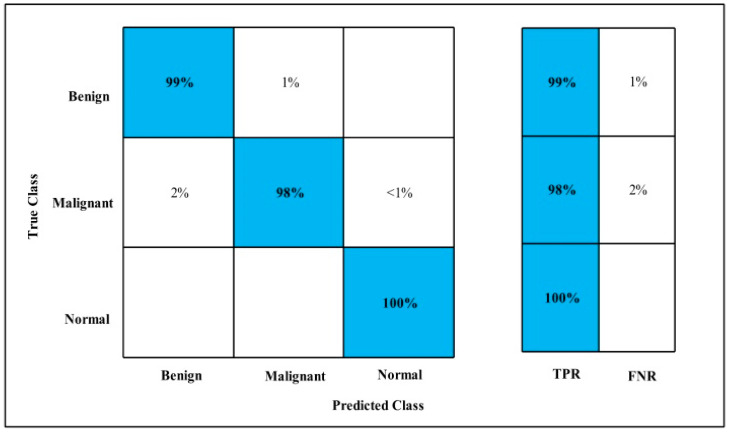
MEWOA on Fine-tuned Nasnet TPR for MIAS.

The second highest accuracy is achieved by Cubic SVM, which is 99.6% in 18.35 (s). Each classifier sensitivity rate is calculated and WKNN has the best value of 99%, which can be confirmed using the confusion matrix shown in Figure 17.

In Table 12, Fusion on Fine-tuned MobilenetV2 and the Nasnet model are applied on the MIAS dataset. The GAP layer extracts deep features from the dataset and fed them to the classifiers. 

The highest accuracy is 99.8%, which is achieved by the Cubic SVM classifier in 133.46 (s). In this table, the minimum time is taken by GN-Bayes, which is 60.069 (s), but the accuracy is 96.4%, which is less than that of Cubic SVM. 

**Figure 18:**
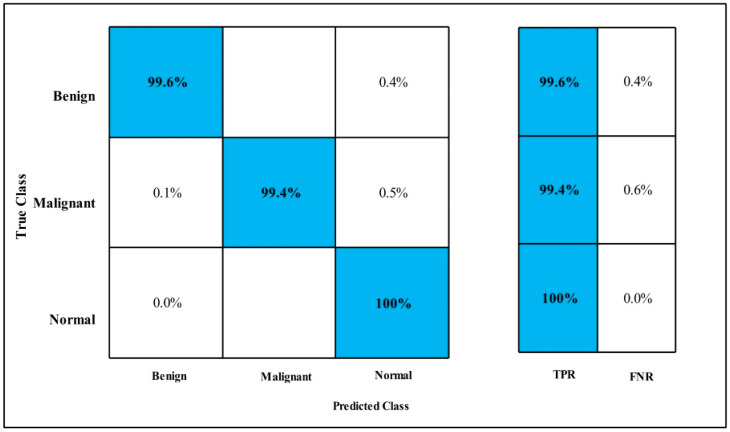
Fusion TPR for MIAS.

The second highest accuracy is achieved by Linear SVM, which is 99.6% in 115.46 (s). Each classifier sensitivity rate is calculated and Cubic SVM has the best value that is 99.66% and can be verified by confusion matrix as described in Figure 18.

In Table 13, MEWOA on Fusion is applied to the MIAS dataset. The deep features of the dataset are extracted from the GAP layer and fed to classifiers. 

The highest accuracy is 99.8%, which is achieved by the Cubic SVM classifier in 63.287 (s). In this table, the minimum time is taken by GN-Bayes, which is 7.9 (s), but the accuracy is 95.7%, which is less than that of Cubic SVM. 

**Figure 19:**
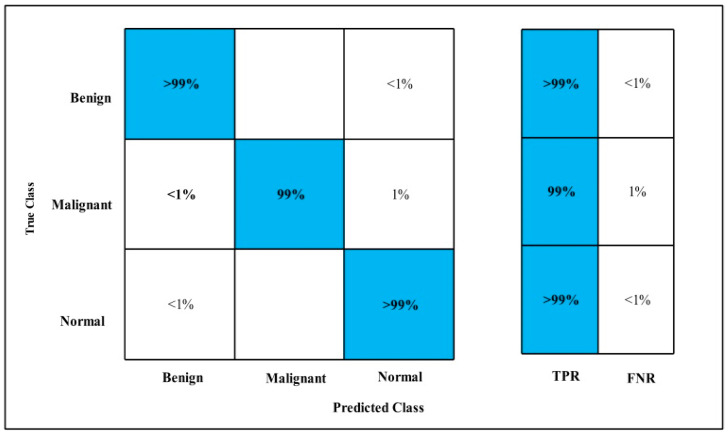
MEWOA on fusion TPR MIAS.

The second highest accuracy is achieved by QSVM, which is 99.7% in 15.37 (s). The sensitivity rate of each classifier is also computed, and the best-noted value for Cubic SVM is 99 %, which can be verified using a confusion matrix, as shown in Figure 19.

**Figure 20:**
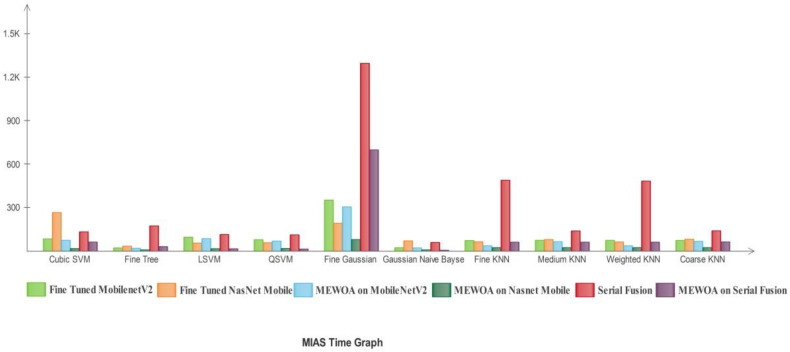
Time compared with individual machine learning classifiers of deep learning models for MIAS.

In Figure 20, the time comparison graph of the models by using the machine learning classifiers is shown. FG-SVM utilized the maximum time in the fusion model. The second highest time was utilized by the FG-SVM classifier in MEWOA serial fusion deep features. GN-Bayes utilized minimum time.

In Table 14, Fine-tuned MobilenetV2 was applied on the INbreast dataset. The GAP layer is used to extract the deep features of the dataset and fed to classifiers. The highest accuracy is 98.3%, which is achieved by the LSVM classifier in 18.80 (s). In this table, the minimum time is taken by GN-Bayes, which is 13.53 (s), but the accuracy is 94.8%, which is less than that of Linear SVM. The second highest accuracy is achieved by QSVM, which is 98.2% in 16.11 (s). 

**Figure 21:**
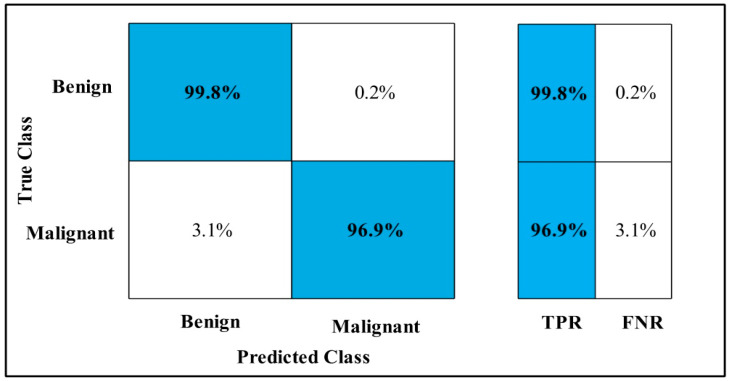
Fine-tuned MobilenetV2 TPR for INbreast.

The sensitivity rate of each classifier is also computed, and the best-noted value is 98.35% for LSVM. It can be confirmed using a confusion matrix, as illustrated in Figure 21.

In Table 15, Fine-tuned Nasnet is applied on the INbreast dataset. The deep features of the dataset are extracted from the GAP layer and fed to classifiers. The highest accuracy is 98.6%, which is achieved by the Cubic SVM classifier in 10.85 (s). In this table, the minimum time is taken by QSVM, which is 9.549 (s), and the accuracy is 98.6%. 

**Figure 22:**
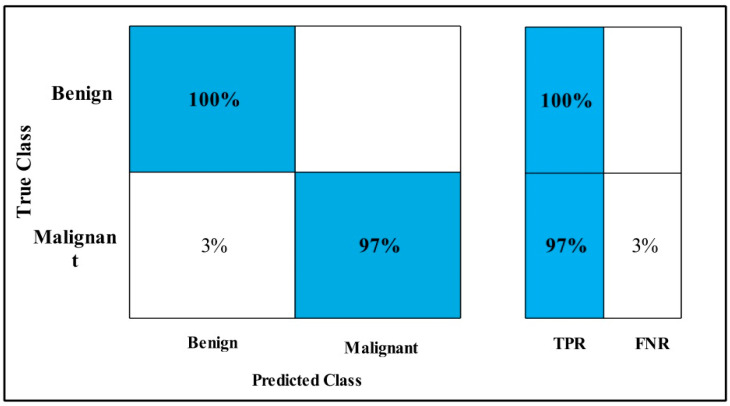
Fine-tuned Nasnet TPR for INbreast.

The second highest accuracy is achieved by GN-Bayes, which is 98.4% in 13.07 (s). Each classifier sensitivity rate is calculated and QSVM has a best-noted value that is 98.5%. The sensitivity rate can be verified by the confusion matrix that is described in Figure 22.

In Table 16, MEWOA was applied on Fine-tuned MobilenetV2 by using INbreast dataset. The deep features of the dataset are extracted from the GAP layer and fed to classifiers. The highest accuracy is 98.3%, which is achieved by the Fine KNN classifier in 35.41 (s). In this table, the minimum time is taken by GN-Bayes, which is 8.4453 (s), and the accuracy is 94.0%.

**Figure 23:**
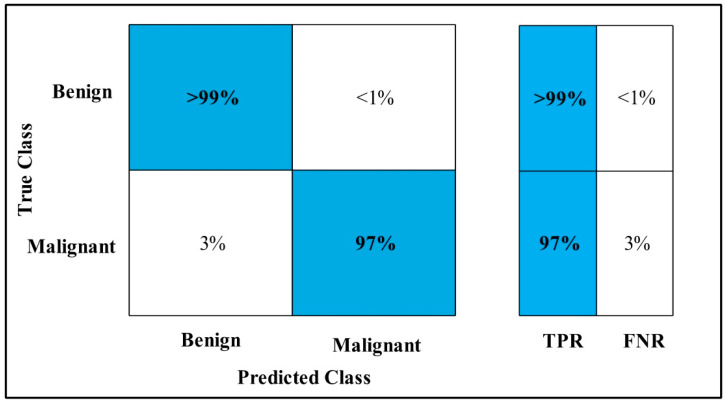
MEWOA on Fine-tuned MobilenetV2 TPR for INbreast.

The second highest accuracy is achieved by QSVM, which is 98.2% in 8.86 (s). The sensitivity rate of each classifier is also computed, and the best-noted value is 98% for Cubic SVM. A confusion matrix, as shown in Figure 23, can be used to verify it.

In Table 17, MEWOA was applied on Fine-tuned Nasnet by using INbreast dataset. The GAP layer is used to extract the deep features of the dataset and features are fed to classifiers. 

The highest accuracy is 98.6%, which is achieved by the Cubic SVM classifier in 6.24 (s). In this table, the minimum time is taken by QSVM, which is 4.55 (s), and the accuracy is 98.6%. 

**Figure 24:**
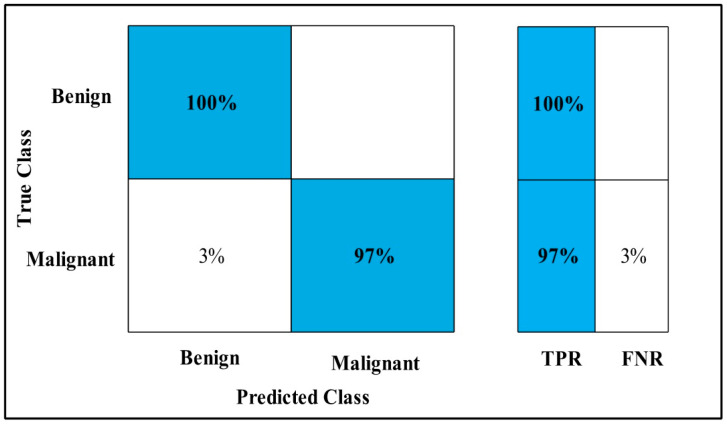
MEWOA on Fine-tuned Nasnet TPR for INbreast.

The second highest accuracy is achieved by WKNN, which is 98.5% in 10.47 (s). The sensitivity rate of each classifier is also computed and Cubic SVM has the best noted value that is 98.5 and It can be verified by using a confusion matrix, as described in Figure 24.

In Table 18, Fusion on MEWOA MobilenetV2 & Nasnet model is applied on the INbreast dataset. The deep features of the dataset are extracted from the GAP layer and fed to classifiers. 

The highest accuracy is 99.9%, which is achieved by the Cubic SVM classifier in 23.04 (s). In this table, the minimum time is taken by Fine Tree, which is 17.63 (s), and the accuracy is 98.8%. 

**Figure 25:**
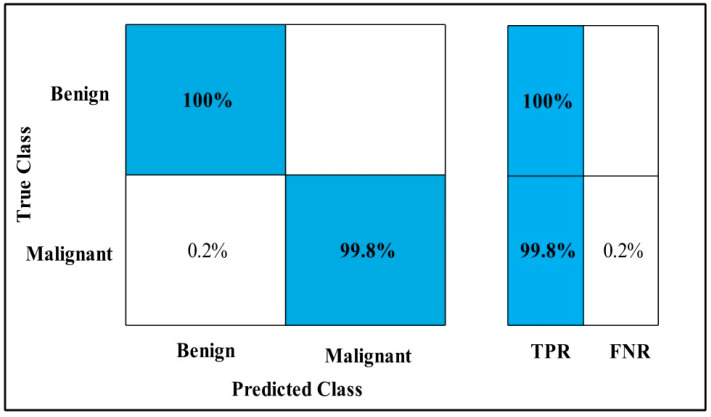
Fusion TPR on INbreast.

The second highest accuracy is achieved by QSVM, which is 99.8% in 23.68 (s). The sensitivity rate of each classifier is also computed, and the best-noted value is 99.9% for Cubic SVM. The confusion matrix is illustrated in Figure 25 to verify the results.

In Table 19, MEWOA on Fusion is applied to the INbreast dataset. The deep features of the dataset are extracted from the GAP layer and fed to classifiers. 

The highest accuracy is 99.7%, which is achieved by the WKNN classifier in 6.57 (s). In this table, the minimum time is taken by Cubic SVM is 1.6178 (s), and the accuracy is 99.1%. 

**Figure 26:**
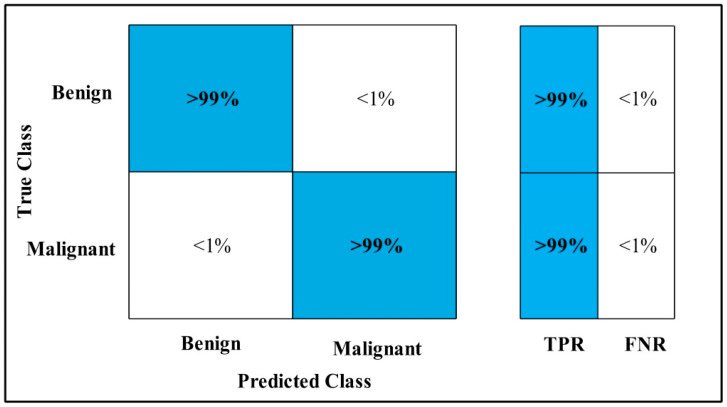
MEWOA on Fusion TPR INbreast.

The second highest accuracy is achieved by Quadratic SVM, which is 99.6% in 1.9933 (s). The sensitivity rate of each classifier is also computed, and the Cubic SVM has a best-noted value that is 99%. A confusion matrix, as shown in Figure 26, can be used to verify the sensitivity rate.

**Figure 27:**
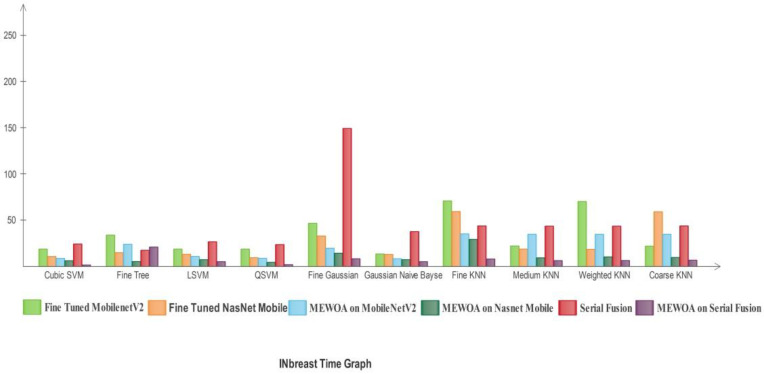
Time compared with individual machine learning classifiers of deep learning models for INbreast.

In Figure 27, the time comparison graph of the deep learning models by using the machine learning classifiers is presented. FG-SVM utilized maximum time in the fusion model. The second highest time was utilized by the Fine KNN classifier in Fine-tuned MobilenetV2. The QSVM utilized minimum time.

Table 20 illustrates comparisons of CBIS-DDSM classification images with respect to other classification studies. The number of images, methods, sensitivity, precision, F1-score, AUC, and accuracy is mentioned in the table. The proposed method shows better results as compared to the other studies.

Table 21 contrasts comparative analysis on MIAS dataset classification images. The number of images, methods, sensitivity, precision, F1-score, AUC, and accuracy compared with the other studies. The proposed method shows good results compared to other studies.

Table 22 shows classification comparisons of the INbreast images with respect to other classification studies. The number of images, methodology, sensitivity, F1-Score, precision, AUC, and accuracy results are mentioned in the table. The proposed method shows good results as compared to the other studies.

## 4. Discussion

Breast cancer for women is a fatal disease all over the world. The women’s life can be saved if cancer is detected at an initial phase. To classify the mammogram images on the basis of features is difficult because optimal features extraction from mammogram images is a challenging task. The three publically available mammogram images dataset of CBIS-DDSM, INbreast, and mini-MIAS are taken to extract the features and perform the classification. Data augmentation is performed to increase the volume of data. Deep learning models achieve best when train with large datasets.The datasets are simulated using the Fine-tuned MobilenetV2 and Nasnet Mobile approaches. To improve the model efficiency the deep features are extracted from the middle layer and fed into MEWOA. The ideal features are selected by using the MEWOA, which reduces the computational cost. The optimal features are selected by using the MEWOA, which reduces the computational cost. The serial fusion is performed by using the MEWOA on MobilenetV2 and MEWOA Nasnet mobile. In the latter, the MEWOA is performed on the fused features to select the best optimized features. The machine learning classifiers are applied. To estimate the performance of the system different measures are applied such as Sensitivity, Precision, F1-Score, AUC, FPR, Accuracy, and Time are computed. All computation is performed on MATLAB2020a by using the personal computer of 16 GB RAM, 4 GB graphics card is used. The comparison time graphs of the figures are made to represent the comparisons of the different classifiers.

The limitation of this proposed approach is that it entails a large volume of the datasets. To rise the size of the datasets, data augmentation is required. The results increased when the data size is large. The deep learning training on a large number of datasets also takes more time. The transfer learning approach is used to increase the efficiency of the system.

## 5. Conclusions

In medical imaging field, extract the features and on the basis of optimized features classification of images is the main domain by using the deep learning procedures. The machine learning classifiers are applied to generate more productive results. This proposed work employed Fine-tuned MobilenetV2 and Nasnet Mobile models to perform the training of the three imbalanced datasets. To extract the deep features the average pool layer is used. Transfer learning and the Adam optimization approaches are utilized to extract the deep features by using the MEWOA on fine-tuned models. The extracted deep features of these optimized models are fused by using the non-redundant serial fusion. The fused deep features are again optimized by using the MEWOA. Finally, classification results are established by applying the machine learning classifiers. The fusion practice increases the accuracy of the results but increases the time of the system. The MEWOM is applied, which optimized the features by reducing the time of computation. By using these techniques, the false-negative and true positive rates decreased. This methodology will be helpful for the radiologists as a second opinion to address the problems of optimal feature extraction and on the basis of optimal features perform the classifications.

## Figures and Tables

**Table 1:** Dataset information.

Dataset	Total Images	Classes	Augmented Images
CBIS-DDSM	1696	2	14,328
INbreast	108	2	7200
MIAS	300	3	14,400

**Table 2:** Classification results using Fine-tuned MobilenetV2 deep features for CBIS-DDSM.

Model	Sensitivity (%)	Precision (%)	F1-Score (%)	AUC	FPR	Accuracy (%)	Time (s)
Cubic SVM	90.20	90.25	90.22	0.96	0.100	90.3	260.13
Fine Tree	72.25	72.50	72.34	0.78	0.275	72.6	30.13
LSVM	77.45	77.55	77.49	0.85	0.225	77.6	296.91
QSVM	85.85	85.95	85.89	0.93	0.140	86.0	287.09
FG-SVM	84.20	88.50	86.29	0.94	0.155	85.2	361.36
GN-Bayes	71.65	71.55	71.59	0.78	0.285	71.5	20.43
FKNN	87.05	86.90	86.97	0.87	0.130	87.0	73.68
MKNN	69.75	70.05	69.89	0.77	0.305	69.2	73.00
WKNN	88.25	88.10	88.17	0.96	0.120	88.2	72.92
Co-KNN	67.45	67.50	67.47	0.75	0.325	67.1	72.90

**Table 3:** Classification results using Fine-tuned Nasnet deep features for CBIS-DDSM.

Models	Sensitivity (%)	Precision (%)	F1-Score (%)	AUC	FPR	Accuracy (%)	Time (s)
Cubic SVM	94.00	94.00	94.00	0.98	0.060	93.9	112.96
Fine Tree	89.50	90.00	89.74	0.93	0.105	89.8	16.91
LSVM	89.50	89.50	89.50	0.96	0.105	89.4	117.51
QSVM	92.50	92.00	92.24	0.98	0.075	92.4	113.75
FG-SVM	84.00	87.50	85.71	0.95	0.160	84.6	275.70
GN-Bayes	84.50	84.50	84.50	0.86	0.155	84.0	18.27
FKNN	93.00	93.00	93.00	0.93	0.010	92.9	60.35
MKNN	87.00	86.50	86.74	0.94	0.130	86.5	60.00
WKNN	94.00	93.50	93.74	0.98	0.060	93.6	59.90
Co-KNN	86.50	86.50	86.50	0.94	0.135	86.4	60.19

**Table 4:** Classification results using MEWOA on MobilenetV2 deep features for CBIS-DDSM.

Models	Sensitivity (%)	Precision (%)	F1-Score (%)	AUC	FPR	Accuracy (%)	Time (s)
Cubic SVM	89.95	90.05	89.99	0.96	0.105	90.0	132.98
Fine Tree	70.20	70.25	70.22	0.76	0.300	70.4	14.83
LSVM	75.45	75.55	75.49	0.83	0.245	75.7	146.36
QSVM	85.15	85.25	85.19	0.92	0.150	85.3	150.17
FG-SVM	83.45	88.05	85.68	0.94	0.165	84.5	178.98
GN-Bayes	70.60	70.50	70.54	0.78	0.295	70.5	8.70
FKNN	86.75	86.60	86.67	0.87	0.130	86.7	37.96
MKNN	69.40	69.55	69.47	0.77	0.305	68.9	37.01
WKNN	87.40	87.35	87.37	0.96	0.125	87.4	37.38
Co-KNN	67.30	67.25	67.27	0.74	0.330	67.3	37.49

**Table 5:** Classification results using MEWOA Nasnet Mobile deep features for CBIS-DDSM.

Models	Sensitivity (%)	Precision (%)	F1-Score (%)	AUC	FPR	Accuracy (%)	Time (s)
Cubic SVM	93.50	93.45	93.47	0.98	0.065	93.5	73.24
Fine Tree	88.95	88.95	88.95	0.92	0.110	89.0	996.56
LSVM	89.15	89.25	89.19	0.96	0.110	89.3	77.09
QSVM	92.25	92.35	92.30	0.97	0.080	92.3	75.26
FG-SVM	84.25	87.60	85.89	0.95	0.160	85.1	182.78
GN-Bayes	84.20	84.45	84.32	0.86	0.160	83.7	11.57
FKNN	92.65	92.50	92.57	0.93	0.075	92.6	40.46
MKNN	86.60	86.40	86.49	0.94	0.135	86.4	39.42
WKNN	93.50	93.45	93.47	0.98	0.065	93.5	42.39
Co-KNN	86.10	86.00	86.04	0.94	0.140	86.1	39.88

**Table 6:** Classification results using Fusion on MobilenetV2 and Nasnet deep features for CBIS-DDSM.

Models	Sensitivity (%)	Precision (%)	F1-Score (%)	AUC	FPR	Accuracy (%)	Time (s)
Cubic SVM	94.10	94.10	94.10	0.99	0.060	94.1	314.97
Fine Tree	88.60	88.65	88.62	0.91	0.115	88.7	151.53
LSVM	92.05	92.05	92.05	0.98	0.080	92.1	271.30
QSVM	93.00	93.05	93.02	0.98	0.070	93.0	265.45
FG-SVM	50.65	76.85	61.05	0.72	0.495	54.0	795.29
GN-Bayes	85.85	86.30	86.07	0.87	0.145	85.5	55.16
FKNN	92.80	92.60	92.69	0.93	0.075	92.6	135.51
MKNN	89.35	89.20	89.27	0.96	0.105	89.2	134.29
WKNN	92.10	92.00	92.04	0.98	0.080	92.1	134.09
Co-KNN	88.60	88.45	88.52	0.96	0.150	88.5	483.34

**Table 7:** Classification results using MEWOA on fusion deep features for CBIS-DDSM.

Models	Sensitivity (%)	Precision (%)	F1-Score (%)	AUC	FPR	Accuracy (%)	Time (s)
Cubic SVM	93.75	93.80	93.77	0.98	0.120	93.8	255.84
Fine Tree	87.90	87.95	87.92	0.90	0.120	88.0	42.42
LSVM	91.75	91.80	91.77	0.98	0.080	91.8	241.52
QSVM	92.90	92.95	92.92	0.98	0.510	93.0	227.28
FG-SVM	50.70	76.85	61.09	0.69	0.450	54.0	692.97
GN-Bayes	85.95	86.00	85.97	0.88	0.140	85.5	59.89
FKNN	92.50	92.45	92.47	0.93	0.075	92.5	408.99
MKNN	88.50	88.40	88.44	0.96	0.115	88.2	83.916
WKNN	92.20	91.75	91.97	0.98	0.085	91.8	407.35
Co-KNN	88.60	88.45	88.52	0.96	0.115	88.5	407.14

**Table 8:** Classification results using Fine-tuned MobilenetV2 deep features for MIAS.

Models	Sensitivity (%)	Precision (%)	F1-Score (%)	AUC	FPR	Accuracy (%)	Time (s)
Cubic SVM	98.73	99.10	98.91	1.00	0.003	99.4	85.29
Fine Tree	79.73	86.03	82.76	0.91	0.933	88.9	22.82
LSVM	96.86	98.20	97.52	1.00	0.013	98.4	97.40
QSVM	98.50	98.96	98.72	1.00	0.003	99.3	79.88
FG-SVM	96.03	98.63	97.31	1.00	0.016	98.2	353.67
GN-Bayes	89.03	81.43	85.06	0.97	0.060	88.5	24.15
FKNN	98.90	98.93	98.91	1.00	0.000	99.4	73.83
MKNN	88.23	89.90	89.05	0.99	0.053	92.6	75.73
WKNN	97.70	98.26	97.97	1.00	0.006	98.8	75.39
Co-KNN	52.06	88.40	65.52	0.96	0.236	77.9	74.30

**Table 9:** Classification results using Fine-tuned Nasnet deep features for MIAS.

Models	Sensitivity (%)	Precision (%)	F1-Score (%)	AUC	FPR	Accuracy (%)	Time (s)
Cubic SVM	99.03	99.13	99.08	1.00	0.000	99.6	267.43
Fine Tree	98.33	98.30	98.31	1.00	0.006	99.1	35.18
LSVM	97.13	98.60	97.86	1.00	0.020	98.7	56.34
QSVM	98.06	98.86	98.46	1.00	0.006	99.1	58.91
FG-SVM	95.83	98.56	97.17	1.00	0.016	98.2	193.88
GN-Bayes	96.00	91.00	93.43	0.98	0.020	95.4	71.71
FKNN	99.03	99.33	99.18	1.00	0.000	99.6	85.83
MKNN	97.63	98.03	97.83	1.00	0.010	98.7	80.99
WKNN	99.20	99.30	99.24	1.00	0.000	99.7	81.45
Co-KNN	96.20	97.8	96.99	1.00	0.01	98.1	82.53

**Table 10:** Classification results using MEWOA on Fine-tuned MobilenetV2 deep features for MIAS.

Models	Sensitivity (%)	Precision (%)	F1-Score (%)	AUC	FPR	Accuracy (%)	Time (s)
Cubic SVM	98.87	98.16	99.01	1.00	0.000	99.4	75.49
Fine Tree	80.00	86.56	83.15	0.91	0.093	89.1	20.09
LSVM	96.77	98.16	97.46	1.00	0.016	98.3	86.78
QSVM	98.73	99.06	98.89	1.00	0.000	99.4	69.90
FG-SVM	95.77	98.66	97.19	0.99	0.016	98.1	305.64
GN-Bayes	89.40	81.80	85.43	0.96	0.056	88.9	22.38
FKNN	98.77	98.83	98.79	0.99	0.006	99.3	65.81
MKNN	88.40	89.50	88.94	0.98	0.056	92.4	65.34
WKNN	97.80	98.40	98.09	1.00	0.006	98.9	64.05
Co-KNN	51.30	88.30	64.89	0.96	0.240	77.6	67.94

**Table 11:** Classification results using MEWOA on Fine-tuned Nasnet deep features for MIAS.

Models	Sensitivity (%)	Precision (%)	F1-Score (%)	AUC	FPR	Accuracy (%)	Time (s)
CSVM	99.00	99.00	99.00	1.00	0.000	99.6	18.35
Fine Tree	97.66	98.00	97.83	1.00	0.003	98.9	9.40
LSVM	97.00	99.00	97.98	1.00	0.010	98.8	17.49
QSVM	98.00	99.00	98.49	1.00	0.006	99.1	18.92
FG-SVM	96.00	98.66	97.31	1.00	0.016	98.2	80.55
GN-Bayes	95.00	90.00	92.43	0.97	0.023	94.9	9.06
FKNN	98.66	98.66	98.66	0.99	0.000	99.6	25.15
MKNN	97.33	97.66	97.49	1.00	0.010	98.5	24.62
WKNN	99.00	99.00	99.00	1.00	0.000	99.7	24.70
Co-KNN	95.66	98.00	96.81	1.00	0.013	98.1	25.10

**Table 12:** Classification results using Fusion deep features for MIAS.

Models	Sensitivity (%)	Precision (%)	F1-Score (%)	AUC	FPR	Accuracy (%)	Time (s)
Cubic SVM	99.66	99.86	99.73	1.00	0.003	99.8	133.46
Fine Tree	98.00	98.00	98.00	0.98	0.006	98.9	175.03
LSVM	99.20	99.73	99.46	1.00	0.006	99.6	115.46
QSVM	99.66	99.90	99.78	1.00	0.000	99.8	113.38
FG-SVM	47.33	91.60	62.05	0.83	0.260	76.2	1276.20
GN-Bayes	97.23	92.70	94.91	0.98	0.016	96.4	60.06
FKNN	99.16	98.96	99.06	0.99	0.003	99.4	490.17
MKNN	98.33	99.00	98.66	1.00	0.020	99.3	140.46
WKNN	98.76	99.46	99.11	1.00	0.003	99.4	484.52
Co-KNN	96.60	98.30	97.44	1.00	0.010	98.7	141.04

**Table 13:** Classification results using MEWOA on Fusion deep features for MIAS.

Models	Sensitivity (%)	Precision (%)	F1-Score (%)	AUC	FPR	Accuracy (%)	Time (s)
Cubic SVM	99.00	99.33	99.16	1.00	0.000	99.8	63.28
Fine Tree	97.20	97.10	97.14	0.99	0.013	98.2	31.91
LSVM	98.76	99.16	98.96	1.00	0.000	99.3	16.55
QSVM	99.43	99.70	99.56	1.00	0.000	99.7	15.37
FG-SVM	47.60	91.60	62.64	0.78	0.260	76.3	699.49
GN-Bayes	96.00	91.66	93.78	0.98	0.020	95.7	7.09
FKNN	99.20	99.20	99.20	0.99	0.003	99.5	62.45
MKNN	98.30	98.53	98.41	1.00	0.006	98.9	62.35
WKNN	98.83	99.40	99.11	1.00	0.003	99.4	62.49
Co-KNN	96.46	98.03	97.24	1.00	0.013	98.2	63.85

**Table 14:** Classification results using Fine-tuned MobilenetV2 deep features for INbreast.

Models	Sensitivity (%)	Precision (%)	F1-Score (%)	AUC	FPR	Accuracy (%)	Time (s)
Cubic SVM	98.20	98.15	98.17	0.99	0.020	98.1	18.89
Fine Tree	97.85	97.75	97.79	0.99	0.020	97.8	34.00
LSVM	98.35	98.25	98.29	1.00	0.015	98.3	18.80
QSVM	98.25	98.20	98.22	0.99	0.015	98.2	16.11
FG-SVM	97.65	97.60	97.62	0.98	0.025	97.6	46.86
GN-Bayes	94.85	94.80	94.82	0.97	0.050	94.8	13.53
FKNN	98.30	98.20	98.24	0.98	0.015	98.2	71.07
MKNN	95.30	95.25	95.27	1.00	0.065	95.1	22.20
WKNN	98.05	98.00	98.02	1.00	0.020	98.0	70.38
Co-KNN	92.70	92.80	92.74	0.99	0.075	92.4	22.05

**Table 15:** Classification results using Fine-tuned Nasnet deep features for INbreast.

Models	Sensitivity (%)	Precision (%)	F1-Score (%)	AUC	FPR	Accuracy (%)	Time (s)
Cubic SVM	98.50	98.50	98.50	1.00	0.0150	98.6	10.85
Fine Tree	98.50	98.50	98.50	1.00	0.0150	98.3	14.92
LSVM	98.50	98.50	98.50	1.00	0.0150	98.6	13.23
QSVM	98.50	98.50	98.50	1.00	0.0150	98.6	9.54
FG-SVM	98.50	98.00	98.24	0.99	0.0150	98.2	33.00
GN-Bayes	98.00	98.00	98.00	0.99	0.0150	98.4	13.07
FKNN	98.50	98.50	98.50	0.99	0.0150	98.6	59.50
MKNN	98.00	98.00	98.00	1.00	0.0150	98.4	18.79
WKNN	98.50	98.00	98.25	1.00	0.0150	98.4	18.53
Co-KNN	98.00	98.00	98.00	1.00	0.020	98.1	59.31

**Table 16:** Classification results using MEWOA on Fine-tuned MobilenetV2 deep features for INbreast.

Models	Sensitivity (%)	Precision (%)	F1-Score (%)	AUC	FPR	Accuracy (%)	Time (s)
Cubic SVM	98	98	98	0.99	0.02	98.1	8.77
Fine Tree	98	98	98	1.00	0.02	97.8	24.05
LSVM	98	98	98	1.00	0.02	98.1	10.92
QSVM	98	98	98	0.99	0.015	98.2	8.86
FG-SVM	97.5	97.5	97.5	0.98	0.025	97.8	19.83
GN-Bayes	94	94	94	0.97	0.06	94.0	8.44
FKNN	98	98	98	0.98	0.015	98.3	35.41
MKNN	94.5	94.5	94.5	1.00	0.055	94.2	34.88
WKNN	98	98	98	1.00	0.02	98.2	34.86
Co-KNN	93.5	93.5	93.5	0.98	0.065	93.4	34.89

**Table 17:** Classification results using MEWOA Fine-tuned Nasnet deep features for INbreast.

Models	Sensitivity (%)	Precision (%)	F1-Score (%)	AUC	FPR	Accuracy (%)	Time (s)
Cubic SVM	98.50	98.50	98.50	1.00	0.0150	98.6	6.24
Fine Tree	98.50	98.50	98.50	1.00	0.0150	98.4	5.44
LSVM	98.50	98.50	98.50	1.00	0.0150	98.6	7.47
QSVM	98.50	98.50	98.50	1.00	0.0150	98.6	4.55
FG-SVM	98.50	98.00	98.24	0.99	0.0150	98.2	14.49
GN-Bayes	98.00	98.00	98.00	0.99	0.0150	98.4	7.47
FKNN	98.50	98.50	98.50	0.99	0.0150	98.6	29.35
MKNN	98.50	98.40	98.44	1.00	0.0150	98.4	9.47
WKNN	98.50	98.50	98.50	1.00	0.0150	98.5	10.47
Co-KNN	98.00	97.50	97.74	1.00	0.0200	97.8	9.75

**Table 18:** Classification results using Fusion deep features for INbreast.

Models	Sensitivity (%)	Precision (%)	F1-Score (%)	AUC	FPR	Accuracy (%)	Time (s)
Cubic SVM	99.90	99.90	99.90	1.00	0.000	99.9	23.04
Fine Tree	99.00	99.0	99.00	0.99	0.010	98.8	17.63
LSVM	99.50	99.50	99.50	1.00	0.000	99.8	26.72
QSVM	99.50	99.50	99.50	1.00	0.000	99.8	23.68
FG-SVM	52.65	76.95	62.52	0.76	0.475	55.0	149.5
GN-Bayes	99.00	99.00	99.00	0.99	0.010	99.1	37.75
FKNN	99.00	99.00	99.00	1.00	0.000	99.6	44.00
MKNN	99.00	99.00	99.00	1.00	0.010	98.9	43.68
WKNN	99.50	99.50	99.50	1.00	0.005	99.5	43.64
Co-KNN	98.50	98.50	98.50	1.00	0.015	98.5	43.93

**Table 19:** Classification results using MEWOA on Fusion deep features for INbreast.

Models	Sensitivity (%)	Precision (%)	F1-Score (%)	AUC	FPR	Accuracy (%)	Time
Cubic SVM	99.00	99.10	99.10	1.00	0.010	99.1	1.61
Fine Tree	98.50	98.50	98.50	0.99	0.015	98.6	21.07
LSVM	99.00	99.00	99.00	1.00	0.005	99.6	5.30
QSVM	99.00	99.00	99.00	1.00	0.000	99.6	1.99
FG-SVM	52.50	77.00	62.43	0.78	0.475	54.9	8.40
GN-Bayes	98.00	98.50	98.24	1.00	0.020	98.2	5.33
FKNN	99.00	99.00	99.00	0.99	0.010	99.4	8.16
MKNN	99.00	99.00	99.00	1.00	0.005	99.4	6.43
WKNN	99.00	99.00	99.00	1.00	0.000	99.7	6.57
Co-KNN	99.00	99.00	99.00	1.00	0.005	99.4	6.95

**Table 20:** Comparisons with state of art CBIS-DDSM dataset.

References	Year	Method	Images	Sensitivity (%)	Precision (%)	F1-Score (%)	AUC (%)	Accuracy (%)
[59]	2021	CNN	1592	92.31	90.00	91.76	0.92	91.2
[24]	2020	Nasnet, MobileNet, VGG, Resnet, Xception	1696	_____	______	85.0	0.84	84.4
[26]	2020	MobilenetV1, MobilenetV2	1696	______	70.00	76.00	______	74.5
[60]	2020	DE-Ada*	_____	______	______	______	92.19	87.05
[23]	2019	VGG, Residual Network	_____	86.10	80.10	______	0.91	______
[61]	2019	DCNN, Alexnet	1696	______	______	______	0.80	75.0
[62]	2018	Deep GeneRAtive Multitask	_____	______	______	______	88.4	89
Proposed Method	2021	MobilenetV2, Nasnet Mobile, MEWOM	1696	93.75	93.80	93.77	0.98	93.8

**Table 21:** Comparisons with the state of the art for the MIAS Dataset.

References	Year	Method	Images	Sensitivity (%)	Precision (%)	F1-Score (%)	AUC (%)	Accuracy (%)
[63]	2021	ResNet-18, (ICS-ELM)	322	______	______	______	_____	98.13
[59]	2021	CNN	322	92.72	94.12	93.58	0.94	93.39
[64]	2020	AlexNet, GoogleNet	68	100, 80	97.37, 94.74	98.3, 85.71	0.98, 0.94	98.53, 88.24
[40]	2019	(MA-CNN)	322	96.00	______	______	0.99	96.47
[65]	2019	DCNN, MSVM	322	______	______	______	0.99	96.90
[66]	2019	Convolutional Neural Network Improvement (CNNI-BCC)	______	89.47	90.71	______	0.90	90.50
Proposed Method	2021	Mobilenet V2 & NasNet Mobile, MEWOA	300	99.00	99.33	99.16	1.00	99.80

**Table 22:** Comparisons with state of art for INbreast Dataset.

References	Year	Method	Images	Sensitivity (%)	Precision (%)	F1-Score (%)	AUC (%)	Accuracy (%)
[63]	2021	ResNet-18, (ICS-ELM)	179	______	______	______	_____	98.26
[59]	2021	CNN	387	94.83	91.23	93.22	0.94	93.04
[38]	2020	Inception ResNet V2	107	______	______	______	0.95	95.32
[60]	2020	De-ada*	______	______	______	______	92.65	87.93
[34]	2017	Transfer learning, Random Forest	108	98.0	70.0	______	_____	90.0
Proposed Method	2021	Fine-tuned MobilenetV2, Nasnet, MEWOM	108	99.0	99.0	99.0	1.00	99.7

## Data Availability

CBIS-DDSM, MIAS and INbreast are publically available data sets and are easily accessible from the website. The INbreast data set was used after the permission from INbreast research group.
